# Numerical study of flow and heat transfer in circular T-shaped junction of different cross-sections

**DOI:** 10.1371/journal.pone.0334236

**Published:** 2025-10-17

**Authors:** Eman Muhammad, Humayoun Shahid, Sheheryar Mohsin Qureshi, Muhammad Babar Ramzan

**Affiliations:** 1 Department of Applied Science, National Textile University, Faisalabad, Punjab, Pakistan; 2 School of Computing, Engineering and Physical Sciences, University of the West of Scotland, Paisley, United Kingdom; 3 School of Engineering and Technology, National Textile University, Faisalabad, Punjab, Pakistan; NED University of Engineering and Technology, PAKISTAN

## Abstract

This study investigates fluid flow and convective heat transfer within a smooth, two-dimensional T-shaped junction using a numerical approach. Simulations were conducted by varying the volumetric flow rate ratio *r* (0.25, 0.5, 0.75, and 1), the Reynolds number *Re* (500 to 2500), the Prandtl number *Pr* (1), and the cross-sectional width ratio *w* (0.5 to 2.5) of the outlet. The fluid dynamics were solved using the vorticity–stream function formulation with a compact upwind finite difference scheme and the Implicit-Explicit (IMEX) method, implemented in MATLAB. Flow behavior was analyzed through streamline and isotherm contours, while local and average Nusselt numbers were computed along the junction walls. The results show that lower *r* values lead to stronger vortex formation and asymmetry in the flow and temperature fields, while *r* = 1 yields symmetric and stable patterns. Increasing *Re* enhances heat transfer and transitions the flow toward unsteady regimes. Similarly, wider outlet configurations (higher *w*) promote recirculation and thermal mixing. This study provides valuable insights into how inlet flow, outlet shape, and fluid characteristics interact to influence heat transfer and flow behavior in a smooth T-shaped junction. It also provides insights that can help improve the design of heat exchangers, microfluidic systems, and industrial piping.

## Nomenclature

**Table:** 

Symbol	Definition
*C*_*p*_	Specific heat capacity at constant pressure ($J\;kg^{-1}K^{-1}$)
*L*	Characteristic length of the inlet (m)
*L*	Characteristic width of the outlet (m)
*M*	Number of discrete points along the ξ-axis
*N*	Number of discrete points along the *η*-axis
Nu	Nusselt number
P	Pressure ($N\;m^{-2}$)
Pr	Prandtl number
Re	Reynolds number
*T*_*C*_	Lower (cold) wall temperature (K)
*T*_*H*_	Higher (hot) wall temperature (K)
*T*_0_	Reference temperature (K)
k	Thermal conductivity ($W\;m^{-1}K^{-1}$)
n	Normal direction to the surface
r	Volumetric flow rate ratio
t	Time (s)
*u*_0_	Average velocity through the inlet (m/s)
u	Velocity component in x-direction (m/s)
v	Velocity component in y-direction (m/s)
w	Cross-sectional width ratio of outlet
*α*	Thermal diffusivity ($m^{2}\;s^{-1}$)
*ρ*	Density of the fluid ($kg\;m^{-3}$)
ν	Kinematic viscosity of the fluid ($m^{2}\;s^{-1}$)
*ω*	Vorticity ($s^{-1}$)
*ψ*	Stream function ($m^{2}\;s^{-1}$)
*θ*	Non-dimensional temperature

## Introduction

Fluid dynamics is crucial for studying liquids and gases as it makes us cognizant of the fluid’s behavior in different situations. Fluid dynamics has applications in many fields like weather prediction, Civil Engineering, Space exploration, and many more [1–3]. One significant area where fluid dynamics plays a vital role is in pipeline systems. Learning about different factors that influence this system is essential. This study aims to optimize the existing system and reduce energy loss. Therefore, understanding the effect of the smoothness of the pipe on heat transfer and fluid flow is essential [4]. Tee-shaped junctions have complex geometry and have many uses in the chemical and petroleum industries. With even a slight change in their geometry, the effects can be dramatic [5].

T-shaped junctions have a wide range of applications. They are commonly used as refrigerant distributors, helping to ensure the even distribution of two-phase flow in heat exchangers [6]. Additionally, T-shaped junctions serve as effective phase separators and are cost-effective alternatives to traditional separators, which can reduce both system costs and space requirements in industrial applications [7]. This geometry is also valuable in microfluidic applications [8], where it can facilitate the creation of bubbles and droplets [9]. In the fields of micro and nano-technology, T-shaped junctions are used to manufacture polymer particles [10]. The performance of junctions is significantly affected by the outlet ratios, highlighting the necessity of examining how various outlet combinations influence downstream flow stability and heat transfer characteristics. In recent years, researchers have demonstrated a growing interest in enhancing the thermal and flow performance of T-junctions, particularly under varying outlet conditions. Smooth-wall designs, in particular, have shown promise for enhancing efficiency and overall functionality in such systems [11,12].

Fluid flow in different junction depends on various factors like the junction’s geometry, the length of the pipes being used and the type of the fluid flowing through the junction. The geometry of junctions, especially their cross-sectional areas, smoothness and angles, has been studied to optimize fluid flow and heat transfer. Ma et al. [13] focused on understanding erosion patterns and optimizing parameters, such as bend diameter ratios and angles, for a more durable and efficient pipeline system. Kada et al. [14] examined the effects of different levels of smoothness in the bends of L-shaped pipes, noting that varying smoothness impacts the upper and lower walls differently. They also observed that heat concentration varies with smoothness of junctions. Zhang et al. [15] studied the flow characteristics of bend pipes with different angles and concluded that the $90^{\circ }$ bend pipe has the most significant impact on fluid acceleration. Downstream of the bend, the centerline velocity fluctuates dramatically for pipes with $60^{\circ },90^{\circ }$, and $120^{\circ }$ angles, while other bending angles exhibit only minor variations. Durst et al. [16] investigated laminar flow through a pipe with a sudden contraction, focusing on flow structure and pressure losses near the contraction. Using both experiments and finite difference simulations, it highlights the influence of 23≤Re≤1213 on velocity profiles and validates numerical predictions with experimental data. Dehkordi. [17] explored multiphase flow with sudden expansions and contractions, focusing on flow behavior, pressure drops and phase distribution. It developed flow meters to estimate volumetric flow rates using pressure data, with validation results obtained through CFD and experiments, which showed good agreement.

Recent studies have increasingly emphasized the integration of experimental and numerical approaches to understand better mixing behavior in complex pipe systems. For instance, Grbčić et al. [18] investigated mixing in a pressurized pipe network comprising two Tee junctions with varying inlet flow ratios, junction spacing, and branching configurations. The experimental data were used to validate two CFD models, a passive scalar model and a multiphase model, which were implemented in OpenFOAM. Both approaches yielded comparable results, although the accuracy of these results was highly sensitive to the turbulent Schmidt number. After calibration, both models accurately captured mixing behavior, with the passive scalar model offering a notable advantage in computational efficiency. Evrim et al. [19] conducted wall-resolved Large-Eddy Simulations (LES) of thermal mixing in horizontal and vertical T-junctions, which were validated against experimental measurements. Their results demonstrated that vertical inflow configurations enhance mixing by promoting unstable stratification. The close agreement between simulation and experimental data reinforces the reliability of validated CFD techniques in capturing detailed flow and thermal structures in junction flows.

Beyond passive mixing, active control strategies have also been examined. Huang et al. [20] used a rotating impeller in a rectangular T-junction, employing LES to analyze its effect on flow behavior, mixing length, and thermal uniformity. Although effective in mitigating thermal stratification, such active devices introduce mechanical complexity and are generally applicable only in systems where such interventions are feasible. At much higher Reynolds numbers, relevant to industrial-scale flows like natural gas pipelines, Tuponosov et al. [21] applied modified Reynolds-Averaged Navier–Stokes (RANS) models to approximate turbulent mixing. These models, however, often require non-physical parameter tuning to align with empirical data. At the other end of the modeling spectrum, Wang et al. [22] employed Direct Numerical Simulation (DNS) to study low-Prandtl-number flows over backward-facing steps. Their analysis revealed the emergence of two dominant unsteady behaviors due to flow recirculations, which significantly affected wall temperature distributions. While buoyancy altered vortex structures and associated time scales, the vortex shedding frequency remained largely unchanged.

Prata et al. [23] investigated laminar flow and heat transfer in an annulus with streamwise-periodic geometry, showing that such configurations can enhance heat transfer up to four-folds compared to uniform ducts, with only moderate pressure drop increases. Patankar et al. [24] analyzed fully developed laminar flow and heat transfer in ducts with streamwise-periodic geometry, enabling a modular analysis that eliminates entrance effects. They experienced strong recirculation zones and significantly enhanced the Nusselt numbers compared to conventional laminar flows, with a clear dependence on the *Re*. Xu et al. [25] investigated thermal mixing in a T-shaped microchannel using a combination of theoretical, experimental, and numerical methods. It found that thermal diffusion dominates at the junction, while both diffusion and convection govern the mixing channel.

In this study, we investigate the combined influence of outlet cross-sectional width ratio (*w*), volumetric flow rate ratio (*r*), Reynolds number (*Re*), and Prandtl number (*Pr*) on laminar fluid flow and heat transfer in a two-dimensional smooth T-shaped junction. The present simulations target the laminar, quasi-two-dimensional regimes where 2D models are shown to capture separation and recirculations reliably in bends and separated channels as we observe from the different studies such as sharply bend channel by Matsumoto et al. [26], also Armaly et al. [27] works on BFS experiment and Kaiktsis et al. [28] works on 2D DNS transition in BFS. We therefore restrict attention to Re below the range where pronounced 3D instabilities are reported for these archetypal flows. Although the current formulation is two-dimensional, it represents the cross-sectional view of a three-dimensional T-junction with circular inlet and outlet channels, as reflected in the title. While CFD has been frequently used to examine the individual effects of *Re* or geometric parameters, the novelty of this work lies in its systematic investigation of the combined effects of *w*,*r*,*Re* and *Pr* in a smooth T-junction configuration. To accurately capture complex T-junction geometries and flow behavior, a curvilinear coordinate system was employed along with a vorticity stream function formulation. Furthermore, a compact upwind scheme was used to enhance the accuracy of the convective term along with the IMEX (Implicit-Explicit) method for time integration, providing higher numerical stability and accuracy. This combination of physical and numerical approaches represents a comprehensive and less commonly reported method in the literature. All simulations and visualizations were carried out using MATLAB. These results help deepen our understanding of how to improve flow behavior and heat transfer in T-junctions, which helps design more efficient heat exchangers, microchannel systems, and other engineering applications.

## 1 Problem formulation

In this section, we will discussed the domain of smooth T-shaped junction, how it is developed and the different parameters that are taken as the part of the problem.

### 1.1 Geometry and problem description

**Fig 1:**
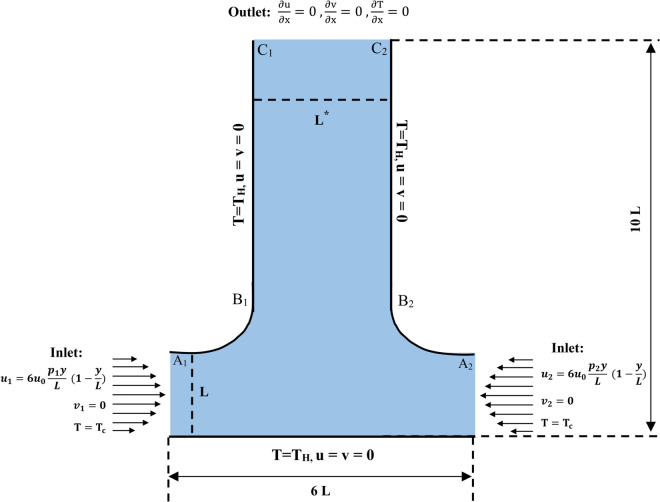
Geometry of Smooth T-shape Junction.

In Fig 1, the two-dimensional geometry of a smooth T-shaped junction is illustrated. This figure has a height of 10*L*, a base width of 6*L*, and a thickness of the pipe of *L* at the inlets and *L*  at the outlet. In our cases *L*  changes. The edges of the T-shaped junctions are smooth without any sharpness. The walls of this junction are heated at *T* = *T*_*H*_ and the fluid entering through the inlet has the temperature *T* = *T*_*C*_, also this fluid is fully developed. From the left inlet the fluid has velocities $u_{1}=6u_{0}\frac{p_{1}y}{L}\left(1-\frac{y}{L}\right)$, $v_{1}=0$ and the right inlet has velocities $u_{2}=6u_{0}\frac{p_{2}y}{L}\left(1-\frac{y}{L}\right)$, $v_{2}=0$. The volumetric flow rate ratio $r=\frac{p_{1}}{p_{2}}$ represents the relative flow rate of the right inlet (*p*_1_) to the left inlet (*p*_2_), with *p*_2_ held constant. The parameters *p*_1_ and *p*_2_ have units of velocity scale factors and they define the values of the inlet velocities. The cross-sectional width ratio of the outlet, denoted as *w*, is given by $w=\frac{L^{*}}{L}$, where *L*  and *L* represent the characteristic width of the outlet and the length of the inlet, respectively. At the outlet, velocity is given by $\frac{\partial u}{\partial y}=0$, $\frac{\partial v}{\partial y}=0$ and temperature is given by $\frac{\partial T}{\partial y}$. There is no slip condition on the walls of the junction, resulting in no velocity along the wall. The reference Reynolds number is defined as $Re=\frac{\rho u_{0}L}{\mu }$. For the inlet profiles given above, the mean velocities are $U_{i}=p_{i}u_{0}$, so the total volumetric flow is $(p_{1}+p_{2})u_{0}L$. Using the outlet width L^*^ = *wL* as the length scale, the corresponding outlet Reynolds number evaluates to $Re_{\text{out}}=Re\;(p_{1}$  +  *p*_2_). Importantly, this expression shows that $Re_{\text{out}}$ is independent of the outlet width ratio *w* and depends only on the total inlet flow level. All the cases reported were checked to ensure $Re_{\text{out}}$ remained within the laminar regime; cases exhibiting unsteadiness are discussed in the Results section. The fluid is incompressible and viscous. The assumption of constant viscosity is widely adopted in classical studies on natural convection, such as in square cavities, L-shaped domains, and branching channels, and this simplification allows direct comparison of the present results with established benchmark cases [29].

### 1.2 Governing equations

The governing equations for the fluid flow and heat transfer are derived from the principle of conservation. The conservation of mass and the conservation of momentum lead to the Navier-Stokes equation whereas the conservation of energy leads to the energy equation which are used for the calculation of fluid flow and heat transfer, respectively [30]. These equations are given as:


$$\frac{\partial u}{\partial x}+\frac{\partial v}{\partial y}=0,$$

$$\frac{\partial u}{\partial t}+u\frac{\partial u}{\partial x}+v\frac{\partial u}{\partial y}=-\frac{1}{\rho }\frac{\partial P}{\partial x}+\nu \left(\frac{\partial^{2}u}{\partial x^{2}}+\frac{\partial^{2}u}{\partial y^{2}}\right),$$

$$\frac{\partial v}{\partial t}+u\frac{\partial v}{\partial x}+v\frac{\partial v}{\partial y}=-\frac{1}{\rho }\frac{\partial P}{\partial y}+\nu \left(\frac{\partial^{2}v}{\partial x^{2}}+\frac{\partial^{2}v}{\partial y^{2}}\right),$$

$$\frac{\partial T}{\partial t}+u\frac{\partial T}{\partial x}+v\frac{\partial T}{\partial y}=\alpha \left(\frac{\partial^{2}T}{\partial x^{2}}+\frac{\partial^{2}T}{\partial y^{2}}\right),$$

where, *u*, *v* are velocities of the fluid in the *x* and *y*–*axis*, *P* is pressure, *ρ* is density of the fluid, ν is viscosity of the fluid, *α* is thermal diffusivity, given as $\alpha =\frac{k}{\rho C_{p}}$ where *k* is thermal conductivity and *C*_*p*_ is specific heat.

The boundary conditions associated with these equations for our problem is given by Table 1.

**Table 1:** Boundary conditions for *u*, *v*, and *T.*

Regions	*u*	*v*	*T*
Left Inlet	$u_{1}=6u_{0}\frac{p_{1}y}{L}\left(1-\frac{y}{L}\right)$	v=0	*T* = *T*_*C*_
Right Inlet	$u_{2}=6u_{0}\frac{p_{2}y}{L}\left(1-\frac{y}{L}\right)$	v=0	*T* = *T*_*C*_
Left wall	*u* = 0	v=0	*T* = *T*_*H*_
Right wall	*u* = 0	v=0	*T* = *T*_*H*_
Bottom wall	*u* = 0	v=0	*T* = *T*_*H*_
Outlet	$\frac{\partial u}{\partial y}=0$	$\frac{\partial v}{\partial y}=0$	$\frac{\partial T}{\partial y}=0$

Converting these equations into dimensionless form with help of non-dimensional variable [31] given by:


$$u^{*}=\frac{u}{u_{0}},\;v^{*}=\frac{v}{u_{0}},\;x^{*}=\frac{x}{L},\;y^{*}=\frac{y}{L},\;t^{*}=\frac{tu_{0}}{L},\;P^{*}=\frac{P}{\rho u_{0}^{2}},\;\theta =\frac{T-T_{0}}{T_{H}-T_{C}}+\frac{1}{2},$$

where, *u*_0_ is the average velocity through the inlet, *L* is the characteristic length of the inlet, *ρ* is the density of the fluid, $T_{0}=\frac{T_{H}+T_{C}}{2}$, *T*_*H*_ is higher temperature whereas *T*_*C*_ is lower temperature. Using these non-dimensional variables in the above equations, we get the equations:

$$\frac{\partial u^{*}}{\partial x^{*}}+\frac{\partial v^{*}}{\partial y^{*}}=0,$$ (1)

$$\frac{\partial u^{*}}{\partial t^{*}}+u^{*}\frac{\partial u^{*}}{\partial x^{*}}+v^{*}\frac{\partial u^{*}}{\partial y^{*}}=-\frac{\partial P^{*}}{\partial x^{*}}+\frac{1}{Re}\left(\frac{\partial^{2}u^{*}}{\partial x^{*2}}+\frac{\partial^{2}u^{*}}{\partial y^{*2}}\right),$$ (2)

$$\frac{\partial v^{*}}{\partial t^{*}}+u^{*}\frac{\partial v^{*}}{\partial x^{*}}+v^{*}\frac{\partial v^{*}}{\partial y^{*}}=-\frac{\partial P^{*}}{\partial y^{*}}+\frac{1}{Re}\left(\frac{\partial^{2}v^{*}}{\partial x^{*2}}+\frac{\partial^{2}v^{*}}{\partial y^{*2}}\right),$$ (3)

$$\frac{\partial \theta }{\partial t^{*}}+u^{*}\frac{\partial \theta }{\partial x^{*}}+v^{*}\frac{\partial \theta }{\partial y^{*}}=\frac{1}{Re\;Pr}\left(\frac{\partial^{2}\theta }{\partial x^{*2}}+\frac{\partial^{2}\theta }{\partial y^{*2}}\right).$$ (4)

*Re* is Reynolds number and *Pr* is Prandtl number. Neglecting the asterisks for simplicity of our work.

### 1.3 Vorticity-stream function formulation

To convert our equations into an equation which is described by the stream function and the vorticity of the fluid instead of traditional velocity components, we use the method of vorticity-stream function formulation. With the help of Eq (2) and Eq (3), we get a singular equation given as:

$$\frac{\partial \omega }{\partial t}+u\frac{\partial \omega }{\partial x}+v\frac{\partial \omega }{\partial y}=\frac{1}{Re}\left(\frac{\partial^{2}\omega }{\partial x^{2}}+\frac{\partial^{2}\omega }{\partial y^{2}}\right),$$ (5)

here *ω* is the vorticity of the fluid. Using Stream functions $u=\frac{\partial \psi }{\partial y}$ and $v=-\frac{\partial \psi }{\partial x}$ in the Eq (4) and Eq (5). Also applying stream function for vorticity given as $\omega =\frac{\partial v}{\partial x}-\frac{\partial u}{\partial y}$, this leads to three new equations:

$$\omega =-\frac{\partial^{2}\psi }{\partial x^{2}}-\frac{\partial^{2}\psi }{\partial y^{2}},$$ (6)

$$\frac{\partial \omega }{\partial t}+\frac{\partial \psi }{\partial y}\frac{\partial \omega }{\partial x}-\frac{\partial \psi }{\partial x}\frac{\partial \omega }{\partial y}=\frac{1}{Re}\left(\frac{\partial^{2}\omega }{\partial x^{2}}+\frac{\partial^{2}\omega }{\partial y^{2}}\right),$$ (7)

$$\frac{\partial \theta }{\partial t}+\frac{\partial \psi }{\partial y}\frac{\partial \theta }{\partial x}-\frac{\partial \psi }{\partial x}\frac{\partial \theta }{\partial y}=\frac{1}{RePr}\left(\frac{\partial^{2}\theta }{\partial x^{2}}+\frac{\partial^{2}\theta }{\partial y^{2}}\right).$$ (8)

### 1.4 Elliptic grid generation

Elliptic grid generation is used to form a structured grid that relates to complex geometries by solving Partial Differential equations (PDEs) like the Poisson equation to analyze the grid point distribution. In this paper, Khattri’s functional is used [32]. This mapping converts our Fig 1 into a computational rectangular grid, which is easier to analyze.

Physical domain can be converted into computational domain with the help of mapping, such as (*x*,*y*) represents a singular point in the physical domain, and by mapping it transforms into (ξ,η) in the computational domain. Similarly the vorticity and stream functions of the physical domain can be converted into computational domain as ω=ω(x,y,t)=ω(ξ,η) and ψ=ψ(x,y,t)=ψ(ξ,η), ignoring the time since the physical domain do not change overtime. Using this transformation to convert the Eqs (6), (7) and (8) into computational variables ξ and *η*. The transformed equation is given by Eqs (9), (10) and (11).

$$q_{1}\;\psi_{\xi \xi }-2q_{2}\;\psi_{\xi \eta }+q_{3}\;\psi_{\eta \eta }+J^{2}\;\nabla^{2}\xi \;\psi_{\xi }+J^{2}\;\nabla^{2}\eta \;\psi_{\eta }=-\omega \;J^{2},$$ (9)

$$\frac{\partial \omega }{\partial t}+\frac{1}{J}\left(\omega_{\xi }\;\psi_{\eta }-\psi_{\eta }\;\omega_{\eta }\right)=\frac{1}{Re}\left[\left(\frac{1}{J^{2}}\right)(q_{1}\;\psi_{\xi \xi }-2q_{2}\;\psi_{\xi \eta }+q_{3}\;\psi_{\eta \eta })+\nabla^{2}\xi \;\omega_{\xi }+\nabla^{2}\eta \;\omega_{\eta }\right],$$ (10)

$$\frac{\partial \theta }{\partial t}+\frac{1}{J}(\psi_{\eta }\;\theta_{\xi }-\psi_{\xi }\;\theta_{\eta })=\frac{1}{RePr}\left[\left(\frac{1}{J^{2}}\right)(q_{1}\;\theta_{\xi \xi }-2q_{2}\;\theta_{\xi \eta }+q_{3}\;\theta_{\eta \eta })+\nabla^{2}\xi \;\theta_{\xi }+\nabla^{2}\eta \;\theta_{\eta }\right],$$ (11)

here,


$$q_{1}=x_{\eta }^{2}+y_{\eta }^{2},\;q_{2}=x_{\xi }x_{\eta }+y_{\xi }y_{\eta },\;q_{3}=x_{\xi }^{2}+y_{\xi }^{2},\;J=x_{\xi }y_{\eta }-y_{\xi }x_{\eta },$$

$$\nabla^{2}\xi =\frac{1}{J^{3}}(q_{1}\;(x_{\eta }\;y_{\xi \xi }-y_{\eta }\;x_{\xi \xi })-2q_{2}\;(x_{\eta }\;y_{\xi \eta }-y_{\eta }\;x_{\xi }\eta )+q_{3}\;(x_{\eta }\;y_{\eta \eta }-y_{\eta }\;x_{\eta \eta })),$$

$$\nabla^{2}\eta =\frac{1}{J^{3}}(q_{1}\;(y_{\xi }\;x_{\xi \xi }-x_{\xi }\;y_{\xi \xi })-2q_{2}\;(y_{\xi }\;x_{\xi \eta }-x_{\xi }\;y_{\xi \eta })+q_{3}\;(y_{\xi }\;x_{\eta \eta }-x_{\xi }\;y_{\eta \eta })).$$

The Eqs (9), (10) and (11) are transformed in curvilinear form which are solved with the help of finite difference schemes, IMEX method and compact upwind scheme.

Using $\nabla^{2}\;\xi$ and $\nabla^{2}\;\eta$ in the two second order elliptic equations given by:


$$q_{1}\;x_{\xi \xi }-2q_{2}\;x_{\xi \eta }+q_{3}\;x_{\eta \eta }=-J^{2}\left(P(\xi ,\eta )\;x_{\xi }+Q(\xi ,\eta )\;x_{\eta }\right),$$

$$q_{1}\;y_{\xi \xi }-2q_{2}\;y_{\xi \eta }+q_{3}\;y_{\eta \eta }=-J^{2}\left(P(\xi ,\eta )\;y_{\xi }+Q(\xi ,\eta )\;y_{\eta }\right),$$

For P(ξ,η)=Q(ξ,η)=0, the equations transform into Laplace’s equation.

$$q_{1}\;x_{\xi \xi }-2q_{2}\;x_{\xi \eta }+q_{3}\;x_{\eta \eta }=0,$$ (12)

$$q_{1}\;y_{\xi \xi }-2q_{2}\;y_{\xi \eta }+q_{3}\;y_{\eta \eta }=0\;$$ (13)

These PDEs need boundary conditions to obtain *x* and *y* which satisfy the domain for the Fig 1. These boundary conditions are as:

$$x(1,\eta ),y(1,\eta )=E_{1},\;x(M,\eta ),y(M,\eta )=E_{2},$$ (14)

$$x(\xi ,1),y(\xi ,1)=E_{3},\;x(\xi ,N),y(\xi ,N)=E_{4}.$$

*M* and *N* represents the number of discrete points for ξ−axis and *η*-axis. $E_{1},E_{2},E_{3}$ and *E*_4_ are the sides associated with the boundaries left, top, right and bottom of the Fig 1.

**Fig 2:**
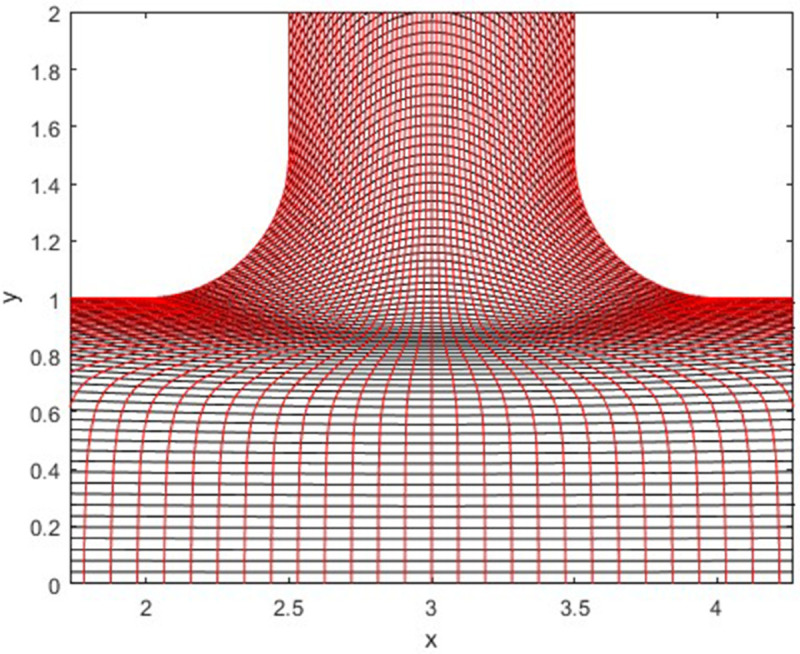
Generated Grid using Elliptic Grid Generation.

The generated grid in Fig 2 is graphed with the help of MATLAB software. This graph is zoomed for more clarification. Furthermore, we used the finite difference method and boundary conditions to solve the Eqs (12) and (13).

**Fig 3:**
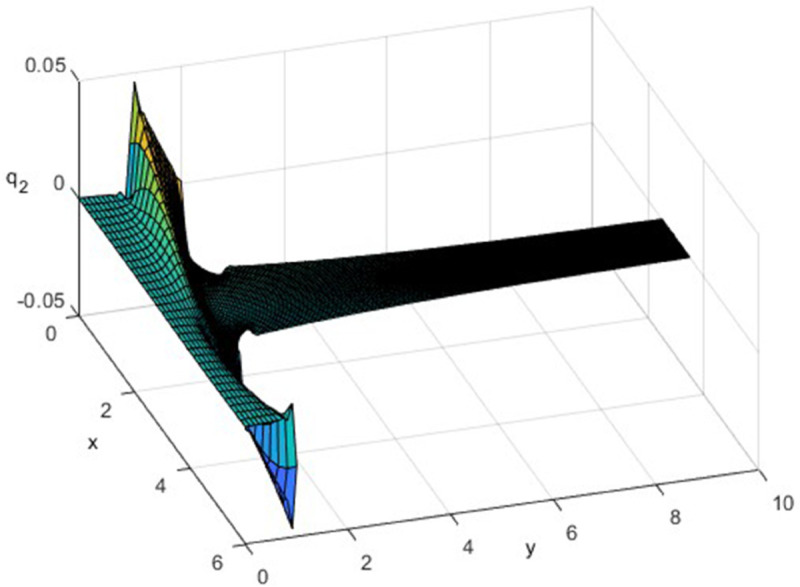
Plotting of $\left(x,y,q_{2}\right)$.

By Fig 3, the cross-metric coefficient *q*_2_, which quantifies the degree of non-orthogonality in the curvilinear grid, is non-zero at the shoulder of the smooth T-shaped junction. Whereas at the outlet channel $q_{2}\approx 0$, which confirms that the grid is orthogonal in that region. Neglecting the non-orthogonality along the shoulders of the junction ensures that the thermal predictions remain unaffected as the Nusselt number is greatly affected by this behavior. Therefore, some skewness may be present near the junction’s shoulder, but it does not influence the overall results of the present study.

### 1.5 Boundary conditions

As our equations are converted into Eqs (9), (10) and (11), we also need to transform our boundary conditions into *ψ*, *ω* and *θ* components for Fig 1. Employing the methodology in [33], we have the revised boundary conditions as given by Table 2.

**Table 2:** Summary of boundary conditions for ψ, ω, and θ.

Regions	ψ	ω	θ
Left Inlet	$3(\frac{\xi }{M}{)}^{2}-2\left(\frac{\xi }{M}\right)$	$6\left(1-2\frac{\xi }{\eta }\right)$	0
Right Inlet	$3(\frac{\xi }{M}{)}^{2}-2\left(\frac{\xi }{M}\right)$	$6\left(1-2\frac{\xi }{\eta }\right)$	0
Left wall	0	$-\frac{q_{1}}{J^{2}}(\psi_{\xi \xi }{)}_{\xi =1}$	1
Right wall	0	$-\frac{q_{1}}{J^{2}}(\psi_{\xi \xi }{)}_{\xi =1}$	1
Bottom wall	0	$-\frac{q_{1}}{J^{2}}(\psi_{\xi \xi }{)}_{\xi =1}$	1
Outlet	${\frac{\partial \psi }{\partial \xi }|}_{\xi =M}=0$	${\frac{\partial \omega }{\partial \xi }|}_{\xi =M}=0$	${\frac{\partial \theta }{\partial \xi }|}_{\xi =M}=0$

The method in [33] is utilized for the second-order accuracy which are to be applied on the conditions of *ω* that have derivatives involved. Second-order Implicit-Explicit (IMEX) method is use for the time discretization of the advection-diffusion equation. Which is given as:

$$\frac{1}{\Delta t}\left[\left(\gamma +\frac{1}{2}\right)u^{n+1}-2\;\gamma \;u^{n}+\left(\gamma -\frac{1}{2}\right)u^{n-1}\right]=(\gamma +1)f(u^{n})-\gamma \;f(u^{n-1})\;+\nu \left[\left(\gamma +\frac{c}{2}\;g(u^{n+1})+(1-\gamma -c)\;g(u^{n})+\frac{c}{2}\;g(u^{n-1})\right)\right]$$ (15)

where we have taken $\gamma =\frac{1}{2}$ and $c=\frac{1}{8}$. We employed multi-grid method to solve the IMEX method, a relevant study can be found in [34].

For Space discretization, we employed the third-order compact upwind scheme for the advection part of the equation. *f*(*u*) represents the advection and *f*(*u*) = *f*(*u*)^ + ^  +  *f*(*u*)^−^. In the study referenced in [35], a third-order upwind compact scheme is proposed, utilizing flux difference splitting to solve the incompressible Navier-Stokes equations. The convection terms in Eq (10) are articulated as follows:


$$\frac{1}{J}\left(\psi_{\eta }\omega_{\xi }-\psi_{\xi }\omega_{\eta }\right)=\frac{1}{J}\left({\left(\psi_{\eta }\omega \right)}_{\xi }+{\left(-\psi_{\xi }\omega \right)}_{\eta }\right),$$

where $(f(\omega ){)}_{\xi }={\left(\psi_{\eta }\omega \right)}_{\xi }$ and $(f(\omega ){)}_{\eta }={\left(-\psi_{\xi }\omega \right)}_{\eta }$. In compact scheme we can


$$(f(\omega ){)}_{\xi }={f\;}^{+}{\;}_{\xi }+{f\;}^{-}{\;}_{\xi }$$

In this context, f(ω) denotes the decomposition of the split flux function into its components, ${f\;}^{+}$ and *f *^−^, which propagate in the positive and negative *x* directions, respectively. This decomposition facilitates the management of wave propagation directionality.

Solving for $({f\;}_{\xi }^{+}{)}_{i}$ and $({f\;}_{\xi }^{-}{)}_{i}$, as

$$\frac{2}{3}(f_{x}^{+}{)}_{i,j}+\frac{1}{3}(f_{x}^{+}{)}_{i-1,j}=\frac{\left(5(f_{i,j}^{+}-f_{i-1,j}^{+})+(f_{i+1,j}^{+}-f_{i,j}^{+})\right)}{6\Delta \xi }$$ (16)

$$\frac{2}{3}(f_{x}^{-}{)}_{i,j}+\frac{1}{3}(f_{x}^{-}{)}_{i-1,j}=\frac{\left(5(f_{i+1,j}^{-}-f_{i,j}^{-})+(f_{i.j}^{-}-f_{i-1,j}^{-})\right)}{6\Delta \xi }$$ (17)

They have iterative relationship with


$$\text{at }i=1:\;{\left(f_{\xi }^{+}\right)}_{i,j}=\frac{-11f_{i,j}^{+}+18f_{i+1,j}^{+}-9f_{i+2,j}^{+}+2f_{i+3,j}^{+}}{6\Delta \xi }$$

$$\text{at }i=1:\;{\left(f_{\xi }^{+}\right)}_{i,j}=\frac{-11f_{i,j}^{+}+18f_{i+1,j}^{+}-9f_{i+2,j}^{+}+2f_{i+3,j}^{+}}{6\Delta \xi }$$

with ${f\;}^{+}=\frac{\partial \psi^{+}}{\partial \eta }\omega$ and ${f\;}^{-}=\frac{\partial \psi^{-}}{\partial \eta }\omega$. The $\frac{\partial \psi^{+}}{\partial \eta }=\frac{\frac{\partial \psi }{\partial \eta }+|\frac{\partial \psi }{\partial \eta }|}{2}$ and $\frac{\partial \psi^{-}}{\partial \eta }=\frac{\frac{\partial \psi }{\partial \eta }-|\frac{\partial \psi }{\partial \eta }|}{2}$. Using the same method, $g(\omega {)}_{\eta }={\left(-\psi_{\xi }\omega \right)}_{\eta }$ can be described by switching the roles of *i* and *j*. The second-order terms are discretized using the central difference formula of order 2. We applied the same technique to solve Eq (11).

### 1.6 Nusselt number

The Nusselt number is a dimensionless quantity that characterizes heat transfer through convection. It can be classified into the local Nusselt number and the average Nusselt number.

The local Nusselt number represents convection at a specific point and is defined by the equation:


$$Nu_{\text{local}}=-\frac{L}{\Delta T}\frac{\partial T}{\partial n},$$

Where *n* denotes the normal direction of the surface.

On the other hand, the average Nusselt number reflects the average convection over a selected region. It is given by the equation:


$$Nu_{\text{avg}}=\frac{1}{L}\int_{C}Nu_{\text{local}}\;ds,$$

Where *C* represents the curve along which the average is being calculated and *L* is the total length of the wall.

## 2 Numerical validity and grid independence test

**Fig 4:**
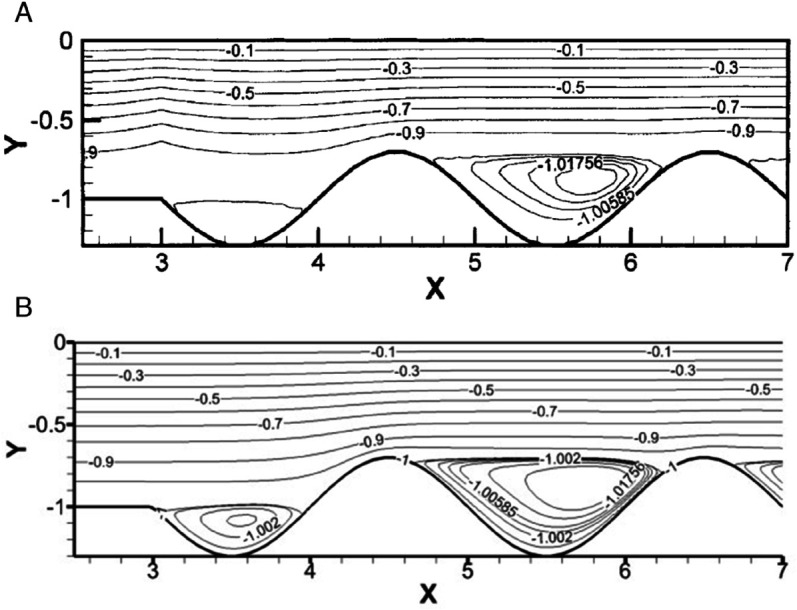
Comparison of Streamlines for flow in wavy channel at Re=500 and α=0.3. **(A)** Shows Wang and Chen [31]. **(B)** Shows our generated model.

To validate our method, we created a wavy channel and applied the same method that we have used in our current geometry, then compared it to the well-established results of Wang and Chen [36]. Their results were generated for *Re* = 500, wave ratio of α=0.3 and their minimum streamfunction value was $\psi_{min}=-1.0119$. The model we generated had the exact boundary conditions and the same values of *Re* and *α*, but we used the vorticity-stream function formulation, whereas Wang and Chen used the simple coordinate transformation method and the spline alternating-direction implicit method. The Fig 4A shows flow in a wavy channel by Wang and Chen [36] whereas Fig 4B shows our created model with the same boundary conditions and values of Wang and Chen’s model.

Table 3, shows the $Nu_{avg}$ along the bottom wall, $\psi_{min}$ and $\psi_{max}$ for different grids. It is observed that the values converge with the increase in the grid size.

**Table 3:** Grid independence test for Re=500, w=1, Pr=1, and r=0.5.

Grid	$Nu_{avg}$ (Bottom Wall)	$Nu_{avg}$ (Left Wall)	$Nu_{avg}$ (Right Wall)	$\psi_{min}$	$\psi_{max}$
17×65	5.8364	4.7865	4.1911	-1.0005	0.5
33×129	6.6749	5.6391	5.4423	-1.0002	0.5
65×257	6.5948	5.7145	5.7166	-1.0003	0.5
129×513	6.6013	5.6848	5.7222	-1.0003	0.5

## 3 Results and discussion

This section presents a comprehensive analysis of fluid flow and thermal behavior under various dimensionless parameters. Simulations were conducted using MATLAB software to investigate the effects of Reynolds number (*Re*), Prandtl number (*Pr*), Volumetric flow rate ratio (*r*) and cross-sectional area of the outlet (*w*) within a T-shaped junction with smooth geometry. The range of parameters are 500≤Re≤2500, *Pr* = 1, r=0.25,0.5,0.75,1 and w=0.5,1,1.5,2, and 2.5. These results are illustrated through streamlines, isotherm and Nusselt number analysis. These simulations help capture the flow, vortices and thermal boundary layer behavior across the range of studied parameters. To provide a focused understanding, several comparisons are made, some based on varying *Re* while keeping *r* and *w* constant, others based on varying *r* or *w* under fixed *Re* and *Pr*, and so on. This structured pairing enables a clear examination of how each dimensionless parameter affects the fluid flow and heat transfer characteristics within the system.

The tolerance of the dimensionless temperature used in the simulations is given by


$$\frac{\left||\theta_{n+1}-\theta_{n}|\right|}{\theta_{n+1}}<1\times 10^{-9}.$$

Similarly, the tolerance for the vorticity is given as


$$\frac{\left||\omega_{n+1}-\omega_{n}|\right|}{\omega_{n+1}}<1\times 10^{-9}.$$

### Influence of *r* on streamlines for *w* = 1.5

**Fig 5:**
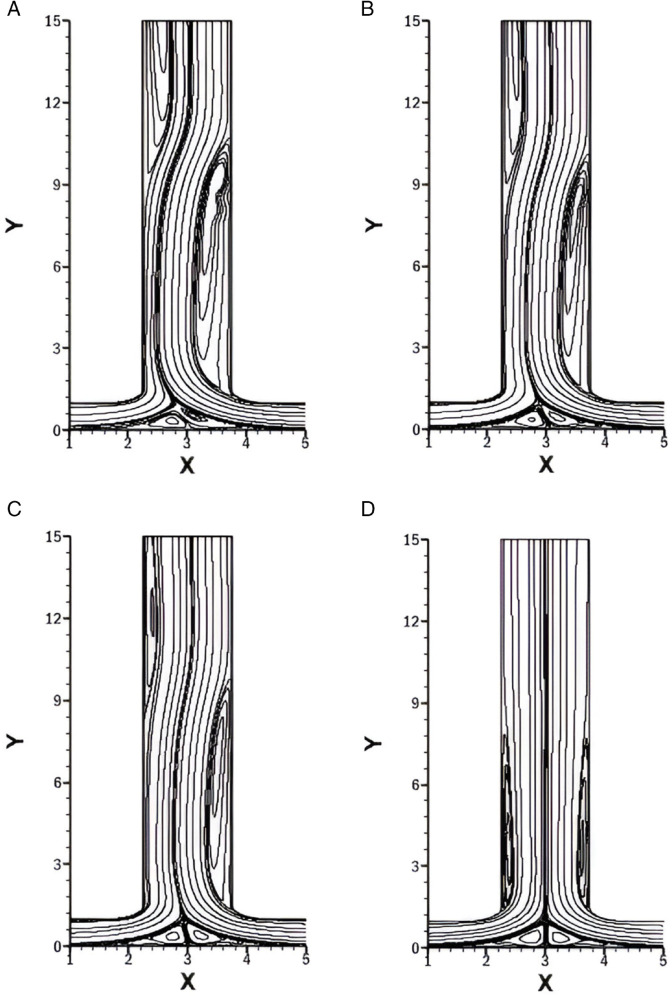
Streamlines for varying *r* with constant w=1.5, Re=1500 and Pr=1. **(A)** Streamlines for *r* = 0.25. **(B)** Streamlines for *r* = 0.5. **(C)** Streamlines for *r* = 0.75. **(D)** Streamlines for *r* = 1.

Fig 5 illustrates the streamlines for different *r*, while keeping the *w* fixed at 1.5, *Re* at 1500, and *Pr* at 1. For *r* = 0.25 as shown in Fig 5A, a strong asymmetry is observed in the flow structure. A pair of vortices appear near the bottom wall, displaced toward the left inlet due to stronger flow from the right inlet. Additionally, a large vortex forms just after the smooth T-shaped junction on the right wall of outlet; this vortex is characterized by width and strength. Due to the high momentum flow from the right inlet, an adverse pressure gradient develops near the right side of outlet, which leads to the formation of a vortex as the flow adjusts to the sudden expansion and changing geometry. This dominant right vortex induces a secondary vortex along the left side of the outlet. The left vortex is also broad but not as intense as the right vortex. This left vortex is formed as a result of flow separation caused by an adverse pressure gradient, leading to a recirculation zone near the walls where fluid flows backward, forming a vortex.

For *r* = 0.5 (Fig 5B), the overall vortex layout remains the same, but their intensity is notably reduced. The bottom wall vortices persist, though less skewed, and both outlet vortices become weaker. The right vortex still dominates, immediately forming after the smooth right bend, while the induced left vortex remains smaller in both strength and width.

With *r* = 0.75 as given in Fig 5C, the intensity of all vortices continues to decrease. The right outlet vortex maintains a relatively consistent length compared to previous cases, but its width is less. The left outlet vortex, however, decreases substantially in both width and length. This shows that the decreasing the flow rate from the right inlet stabilizes the outlet flow, suppressing secondary vortex formation.

For Fig 5D, *r* = 1, the flow rate ratio is symmetric. Identical vortices are observed at the bottom wall and each outlet wall, symmetrically placed just after the respective bends of the T-junction. These outlet vortices are narrower and shorter than those in previous cases, suggesting a more stable and less disturbed flow field due to the balanced inlet momentum.

### Influence of *Re* on streamlines for *r* = 0.25

**Fig 6:**
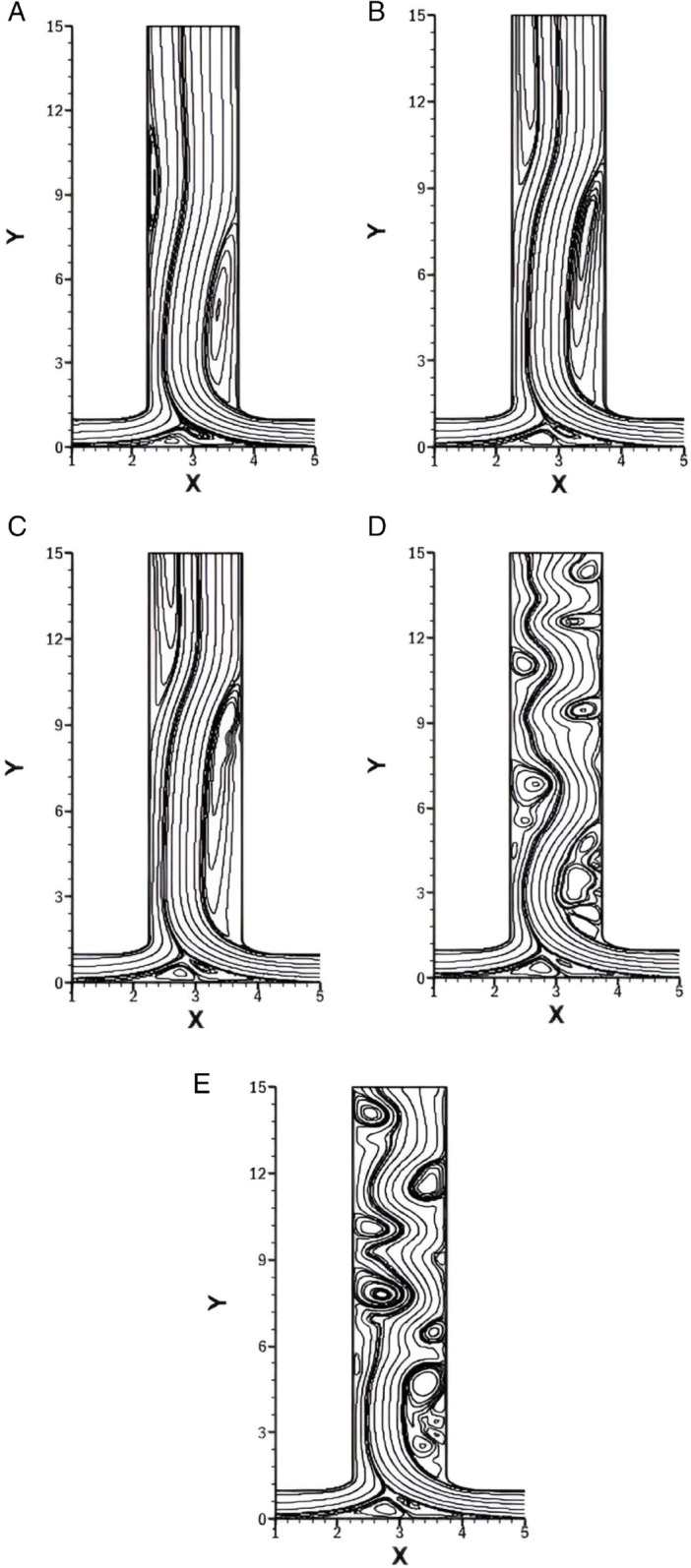
Streamlines for varying *Re* with constant w=1.5, r=0.25 and Pr=1. **(A)** Streamlines at *Re* = 500. **(B)** Streamlines at *Re* = 1000. **(C)** Streamlines at *Re* = 1500. **(D)** Streamlines at *Re* = 2000. **(E)** Streamlines at *Re* = 2500.

In Fig 6, streamlines for varying *Re* ranging from 500 to 2500 with fixed *w* = 1.5, *r* = 0.25 and *Pr* = 1 are presented. Since *r* = 0.25, the right inlet dominates the flow and pushes the fluid towards the left side. As a result, the vortex near the bottom wall is consistently shifted toward the left inlet across all sub-figures of Fig 6.

In Fig 6A, which illustrates *Re* = 500, a vortex is formed immediately after the right bend of the smooth T-shaped junction. While its axial length is relatively short compared to higher *Re* cases, its width remains nearly the same in all the cases. This is due to constant *r*. Additionally, a small secondary vortex emerges at the left side of the outlet, though it remains weak and barely noticeable at low *Re*.

For *Re* = 1000, Fig 6B is illustrated. Here, the vortex that appears on the right side of the outlet becomes more elongated, indicating a stronger recirculation due to increased *Re*. The left-side vortex, located along the outlet, also becomes more pronounced compared to *Re* = 500.

For *Re* = 1500 (Fig 6C), the same trends continues but they are more pronounced. As the inertial forces increase, it allows the fluid to flow further into the outlet channel, making the vortices more strong and longer. The secondary vortex at the left side becomes more distinct, indicating that a stronger primary vortex induces a stronger secondary vortex.

When *Re* is increased to 2000 and 2500 as shown in Fig 6D and Fig 6E, the flow exhibits an unsteady behavior. The vortices become more complex and dynamic, indicating the formation of turbulent flow regimes. The increased *Re* reduces the relative influence of viscous effects, allowing fluctuations and oscillations to grow within the shear layers.

### Influence of *Re* on streamlines for *r* = 0.75

**Fig 7:**
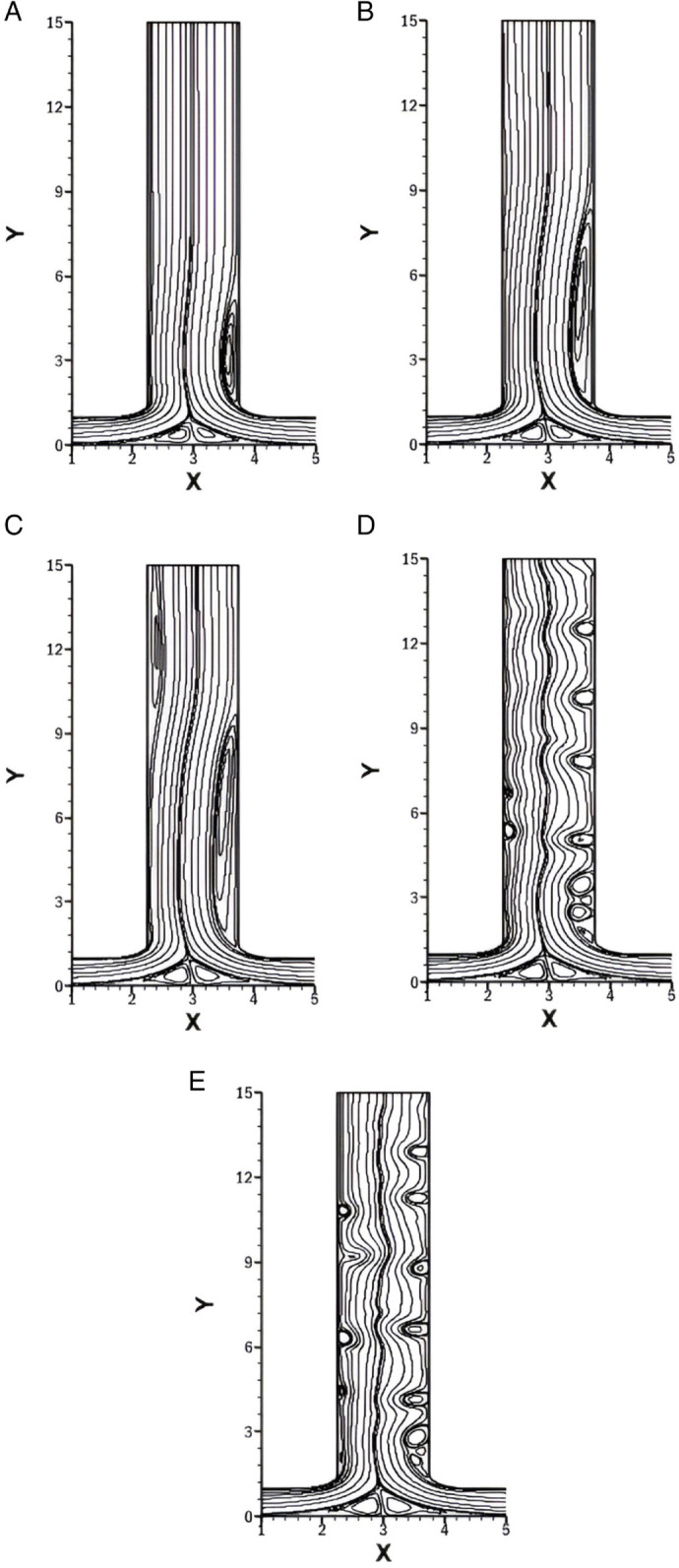
Streamlines for varying *Re* with constant w=1.5, r=0.75 and Pr=1. **(A)** Streamlines at *Re* = 500. **(B)** Streamlines at *Re* = 1000. **(C)** Streamlines at *Re* = 1500. **(D)** Streamlines at *Re* = 2000. **(E)** Streamlines at *Re* = 2500.

Fig 7 display the streamlines for varying *Re* at constant *w* = 1.5, *r* = 0.75 and *Pr* = 1. In this configuration, for *r* = 0.75, the dominance of the right inlet is reduced compared to *r* = 0.25, resulting in a more balanced flow distribution between the two inlets. As a result, the bottom wall vortices are not strongly pushed toward the left inlet as compared to Fig 6.

In Fig 7A *Re* is taken 500, a primary vortex forms on the right side of the outlet just after the smooth bend. The size and width of this vortex are relatively small, and no secondary vortex is observed at this low *Re*.

For Fig 7B, *Re* increases to 1000, the bottom wall vortex remains similar to the one observed in Fig 7A, but the primary vortex at the right side increases slightly in both length and width. Despite the growing strength of the recirculation due to increased *Re*, no secondary vortex is formed, as the flow inertia is not high enough to destabilize the shear layers significantly.

As *Re* increases to 1500, as given in Fig 7C, the same general bottom vortex structure is retained. However, the right-side primary vortex grows significantly in length, but the width is the same as in Fig 7B. A secondary vortex also appears on the left side of the outlet channel.

For *Re* equals 2000 and 2500 as shown in Fig 7D and Fig 7E respectively, the flow becomes unsteady, although it is less intense than Fig 6D and Fig 6E. The size and the strength of the vortices are relatively smaller and the oscillations are less energetic compared to Fig DE.

### Influence of *Re* on streamlines for *w* = 0.5

**Fig 8:**
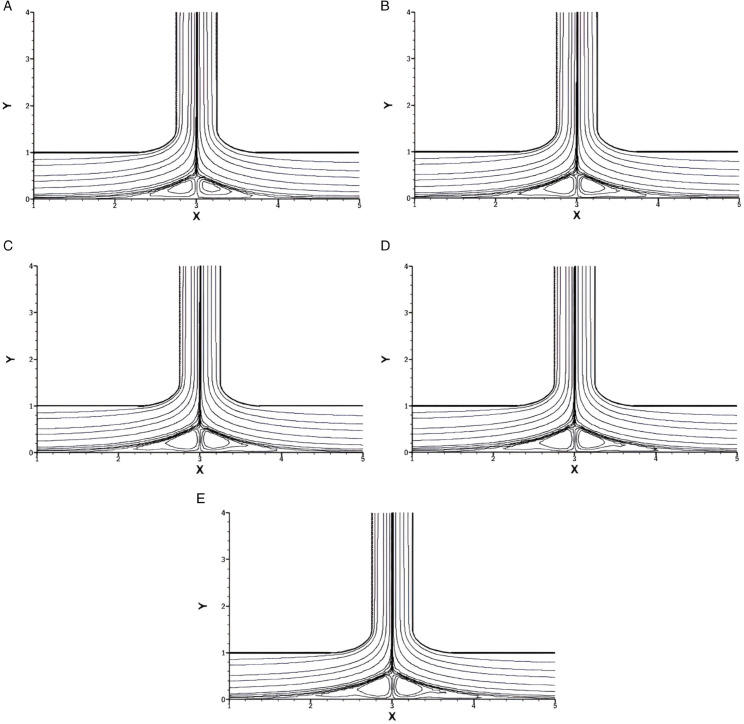
Streamlines for varying *Re* with constant w=0.5, r=1 and Pr=1. **(A)** Streamlines at *Re* = 500. **(B)** Streamlines at *Re* = 1000. **(C)** Streamlines at *Re* = 1500. **(D)** Streamlines at *Re* = 2000. **(E)** Streamlines at *Re* = 2500.

Fig 8 illustrates the streamlines for varying *Re* equals 500, 1000, 1500, 2000 and 2500 with fixed *w* = 0.5, *r* = 1 and *Pr* = 1. The outlet width here is significantly smaller compared to the inlets. A smaller *w* implies a constricted outlet channel, which causes the fluid to accelerate as it exits. This resembles a jet-like flow.

This high-speed ejection and constriction, restricts the formation of recirculation zones along the outlet walls. As a result, no vortices are observed along the outlet walls for any value of *Re*. The only observable changes occur near the bottom wall of the T-shaped junction, where weak recirculating structures form.

As the Re increases from 500 to 2500 as shown in Fig 8A-Fig 8E, these bottom wall vortices exhibit a slight growth in size, which is a due to increased inertial forces.

### Influence of *w* on streamlines at *r* = 0.5

**Fig 9:**
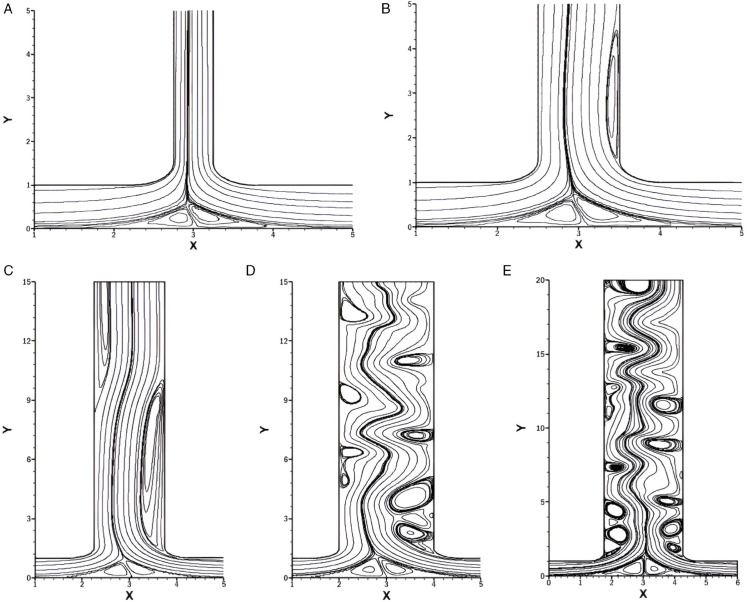
Streamlines for varying *w* with constant Re=1500, r=0.5 and Pr=1. **(A)** Streamlines at *w* = 0.5. **(B)** Streamlines at *w* = 1. **(C)** Streamlines at *w* = 1.5. **(D)** Streamlines at *w* = 2. **(E)** Streamlines at *w* = 2.5.

Fig 9 presents the streamlines for varying *w* with constant *Re* = 1500, *r* = 0.5 and *Pr* = 1. The values of *w* are 0.5, 1. 1.5, 2 and 2.5.

For *w* = 0.5 (Fig 9A), the outlet is significantly narrower than the inlets, creating a jet-like discharge. The high-speed outflow prevents recirculation near the outlet walls, and thus, no vortices are observed in the outlet region. The only vortices appear near the bottom wall of the T-junction and are pushed toward the left inlet due to the imbalance in flow from the inlets caused by *r* = 0.5.

In Fig 9B, *w* is taken as 1, the outlet’s width becomes equal to the inlet length. The bottom wall vortices remain in the same position as in Fig 9A. A small vortex appears along the right side of outlet, formed due to mild boundary layer separation, but no secondary vortex is present.

Fig 9C has *w* = 1.5, and the outlet is wider than the inlets. The primary vortex formed at the right side immediately after the bend of the smooth T-shaped junction has significantly increased in length, indicating stronger flow separation. Additionally, a secondary vortex is also found on the left side of the outlet region.

In Fig 9D, for *w* = 2, the outlet is twice as wide as the inlet length. The wide geometry and high inertial flow promote complex interactions with the boundary layer, which lead to unsteady behavior.

In Fig 9E, the *w* is increased further to 2.5. The fluid becomes unsteady with increased oscillations and larger vortices compared to Fig 9D. The mismatch between the inlets and outlet geometries leads to highly disturbed flow.

To present the qualitative flow behavior observed in the streamlines plots more clearly, the following tables summarize the dominant flow regimes for different parameters. Tables 4–8) represents the corresponding figures and highlights the notable flow structures and key characteristics.

**Table 4:** Flow regimes for different *r* with fixed w=1.5, Re=1500, and Pr=1 (see Fig 5).

Case	*r*	Flow Regime	Key Characteristics
Fig 5A	0.25	Steady asymmetric	Strong right vortex and weaker left vortex. Bottom vortices shifted toward the left inlet.
Fig 5B	0.50	Steady asymmetric	Reduced vortex intensity, but the right vortex is still dominant.
Fig 5C	0.75	Steady weakly asymmetric	Further reduced vortex size and more stable outlet flow.
Fig 5D	1.00	Steady symmetric	Identical and narrow vortices on both outlet walls; balanced inlet momentum.

**Table 5:** Flow regimes for varying *Re* with fixed w=1.5, r=0.25, and Pr=1 (Fig 6).

Case	Re	Flow Regime	Key Characteristics
Fig 6A	500	Steady asymmetric	Short right vortex and weak left vortex, bottom vortex shifted left.
Fig 6B	1000	Steady asymmetric	The right vortex is elongated, whereas the left vortex is more distinct.
Fig 6C	1500	Steady asymmetric	Stronger and longer vortices, also pronounced secondary left vortex.
Fig 6D	2000	Unsteady transitional	Complex vortex structure, furthermore, the onset of oscillations.
Fig 6E	2500	Unsteady/turbulent	Highly dynamic vortices and strong fluctuations in shear layers.

**Table 6:** Flow regimes for varying *Re* with fixed w=1.5, r=0.75, and Pr=1 (Fig 7).

Case	Re	Flow Regime	Key Characteristics
Fig 7A	500	Steady weakly asymmetric	Small right vortex and no secondary vortex.
Fig 7B	1000	Steady weakly asymmetric	Slightly larger right vortex and bottom vortex are unchanged.
Fig 7C	1500	Steady weakly asymmetric	Longer right vortex and secondary left vortex emerge.
Fig 7D	2000	Unsteady transitional	Smaller and weaker unsteady vortices than the *r* = 0.25 case.
Fig 7E	2500	Unsteady transitional	Low-intensity oscillations and less energetic than Fig 6E.

**Table 7:** Flow regimes for varying *Re* with fixed w=0.5, r=1, and Pr=1 (Fig 8).

Case	Re	Flow Regime	Key Characteristics
Fig 8A	500	Steady	Narrow outlet accelerates flow, producing a high-speed jet. There are no vortices along the outlet walls and a small stable recirculation near the bottom wall.
Fig 8B	1000	Steady	Flow remains stable. The bottom wall vortex grows slightly in size due to increased inertia and still no outlet wall separation.
Fig 8C	1500	Steady	Slightly stronger bottom wall recirculation. Also, the outlet remains free of large-scale vortices; the flow remains symmetric.
Fig 8D	2000	Steady	Bottom wall vortex increases slightly in size, and the outlet jet still dominates with no major separation zones.
Fig 8E	2500	Steady	Recirculation near the bottom wall is slightly larger, and the outlet still maintains a jet-like flow.

**Table 8:** Flow regimes for varying *w* with fixed Re=1500, r=0.5, and Pr=1 (Fig 9).

Case	w	Flow Regime	Key Characteristics
Fig 9A	0.5	Steady	No outlet vortices and bottom vortices shifted toward the left inlet. The flow is jet-like.
Fig 9B	1.0	Steady asymmetric	Small right vortex, no secondary vortex and bottom vortices unchanged.
Fig 9C	1.5	Steady asymmetric	Long right vortex and secondary left vortex appears.
Fig 9D	2.0	Unsteady transitional	Wide outlet induces unsteady flow.
Fig 9E	2.5	Unsteady/turbulent	Large vortices, strong oscillations and highly disturbed flow.

### Influence of *r* on isotherms for *w* = 1.5

**Fig 10:**
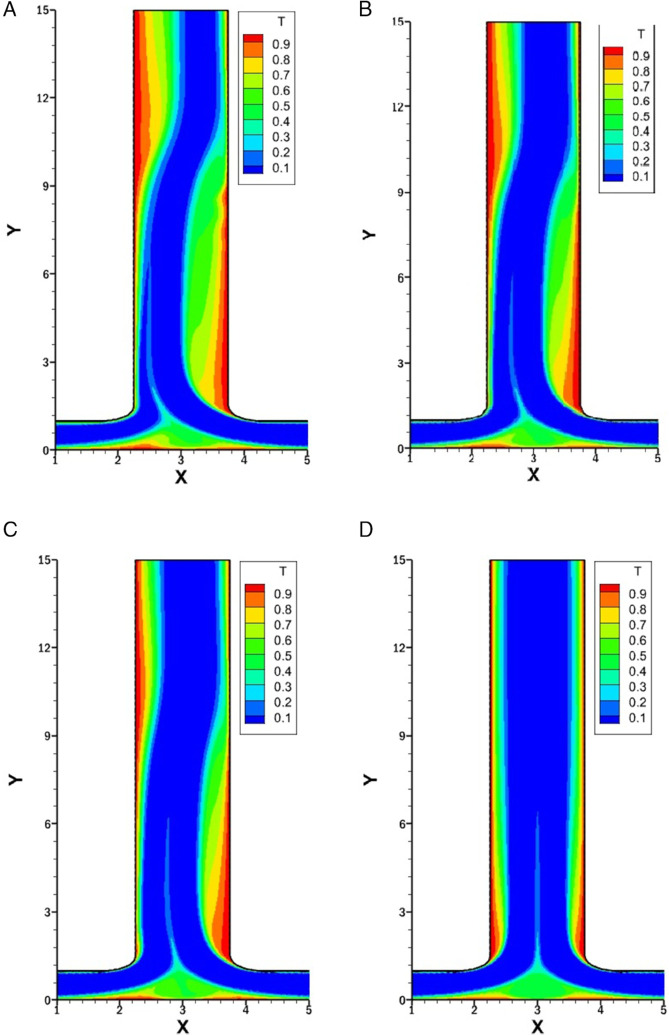
Isotherms for varying *r* with constant w=1.5, Re=1500 and Pr=1. **(A)** Isotherm for *r* = 0.25. **(B)** Isotherm for *r* = 0.5. **(C)** Isotherm for *r* = 0.75. **(D)** Isotherm for *r* = 1.

Fig 10 represents the isotherms for varying *r* of 0.25, 0.5, 0.75 and 1 with constant *w* at 1.5, *Re* at 1500 and *Pr* at 1.

In Fig 10A (*r* = 0.25), the strong inflow from the right inlet produces a prominent vortex on the right side of the outlet, which disrupts the thermal boundary layer and increases the heat transfer. At the start of the left side of the outlet, a stable and thick thermal boundary layer is observed, which corresponds to weak heat transfer. Further downstream, a secondary vortex forms, which again disturbs the boundary layer and increases the heat transfer. After the vortex dissipates, the thermal boundary layer thickens along the right side while it remains thin along the left side. Along the bottom wall, as the flow from two inlets merges near the center, a vortex is created, resulting in a disturbance of the thermal boundary layer and leading to enhanced heat transfer.

In Fig 10B (*r* = 0.5), the flow from the right inlet is weaker compared to the case with *r* = 0.25, resulting in a less intense vortex at the right side of outlet. Consequently, the thermal boundary layer disruption is milder, and the local heat transfer enhancement is reduced. The left wall of outlet initially exhibits a relatively thicker thermal boundary layer, but due to weaker vortex formation downstream, a moderate increase in heat transfer is observed. At the bottom wall, a less intense vortex near the center is observed compared to *r* = 0.25, leading to a noticeable but reduced heat transfer.

In Fig 10C (*r* = 0.75), the flow from both inlets becomes more balanced, and the strength of vortices along the outlet walls decreases further. This leads to less heat transfer and thicker thermal boundary layers compared to Fig 10A. Also the heat transfer along the bottom wall reduces and become more balanced.

In Fig 10D (*r* = 1), the symmetry in inlet flow leads to a symmetric temperature distribution across the domain. The isotherms along both outlet walls are uniform and symmetric. The thermal boundary layers remain stable and relatively thick due to the presence of weak vortices along the outlet walls. As a result, heat transfer is more evenly distributed but lower in intensity compared to previous cases. The bottom wall exhibits symmetrical isotherms centered around the junction, with minimal disturbance in the thermal boundary layer.

### Influence of *Re* on isotherms for *r* = 0.25

**Fig 11:**
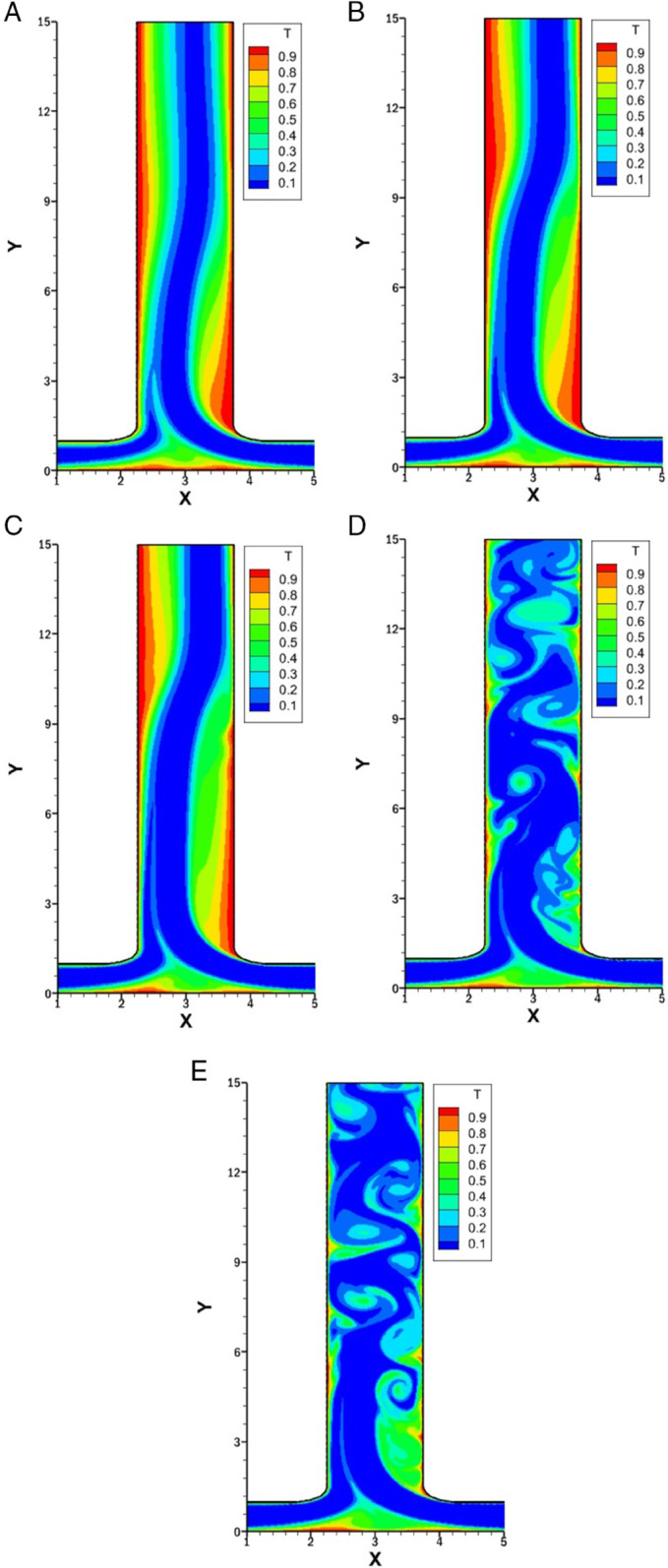
Isotherms for varying *Re* with constant w=1.5, r=0.25 and Pr=1. **(A)** Isotherm at *Re* = 500. **(B)** Isotherm at *Re* = 1000. **(C)** Isotherm at *Re* = 1500. **(D)** Isotherm at *Re* = 2000. **(E)** Isotherm at *Re* = 2500.

Fig 11 represents the isotherms for varying *Re* with constant *w* at 1.5, *r* at 0.25 and *Pr* at 1. The impact of increasing *Re* on thermal boundary layers and heat transfer performance along the outlet and bottom walls is evident across the subfigures.

In Fig 11A *Re* = 500 is taken, at this low *Re*, the thermal boundary layer is well defined. There is no vortex along the left side of the outlet, resulting in a smooth flow, which in turn leads to a thicker thermal boundary layer and lower heat transfer. On the right side of the outlet, a small vortex is present, which leads to a thin thermal boundary layer and enhanced heat transfer. However, the size of this vortex is relatively small. As it dissipates, the thermal boundary layer thickens and heat transfer decreases. Along the bottom wall, weak recirculation results in moderate thermal mixing.

Fig 11B represents the isotherm for *Re* = 1000. As *Re* increases, the intensity and elongation of the vortex present along the right side of the outlet intensify, leading to the thinning of the thermal boundary layer and an increase in heat transfer. On the left side, a very weak vortex is observed, which slightly disturbs the thermal layer boundary and enhances heat transfer along the left side of the outlet. The merging flow at the bottom creates more prominent vortices, which increases the mixing and heat transfer in that region.

Fig 11C illustrates the isotherm for *Re* = 1500. Both the vortices along the outlet become more developed. On the left side, the thermal boundary layer is initially thick and then becomes thin, resulting in enhanced heat transfer. On the right side, the thermal boundary layer is initially thin, and heat transfer is enhanced; however, it becomes thicker afterward. The bottom wall has almost the same thickness of the thermal boundary layer as in previous cases.

Fig 11D and Fig 11E display the isotherms for *Re* at 2000 and 2500, respectively. At this high *Re*, the flow exhibits unsteady behavior, as evident from the irregularity of the isotherms. In both cases, prominent vortices are present at multiple places on both the left and right sides of the outlet channel. These vortices stir up the flow and lead to stronger thermal mixing, which helps thin the thermal boundary layer and boost heat transfer. Although unsteady flow can make the analysis more challenging, it also offers clear advantages by improving mixing and making heat transfer more effective.

### Influence of *Re* on isotherms for *r* = 0.75

**Fig 12:**
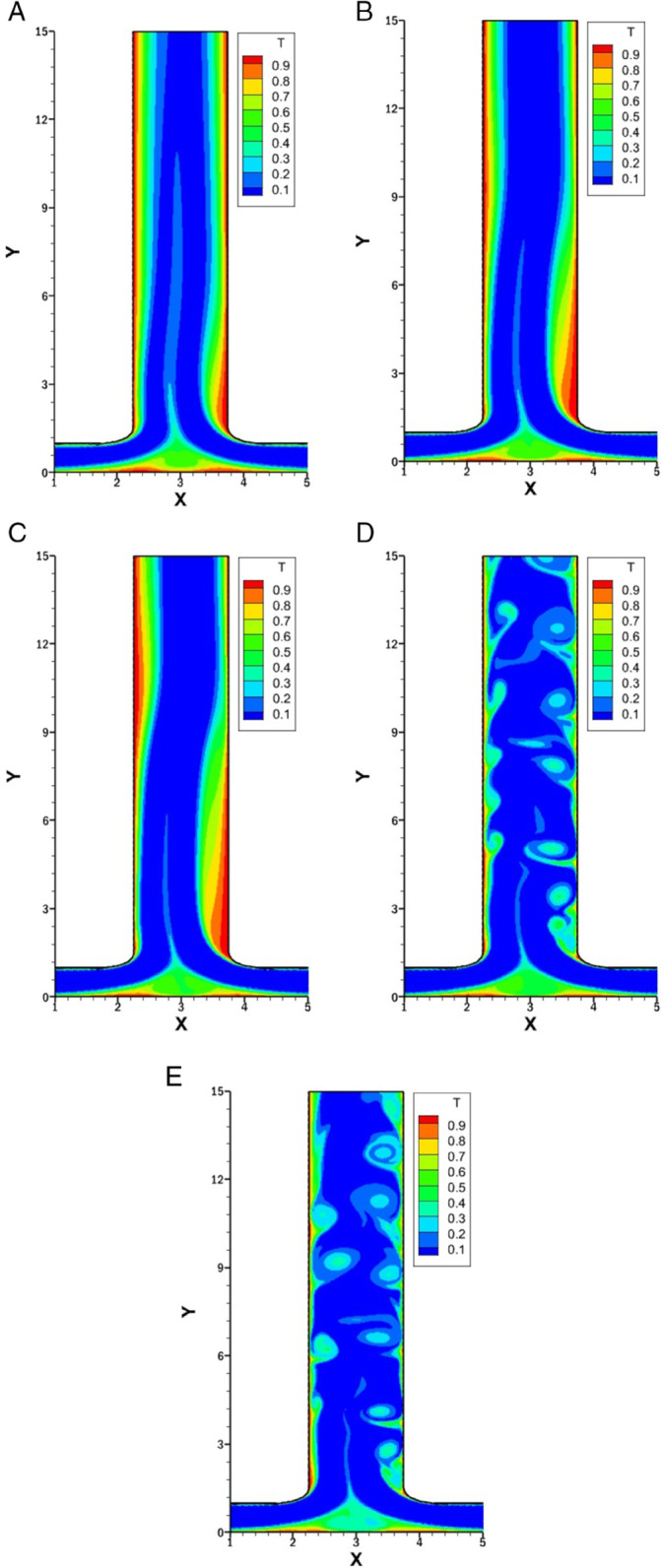
Isotherms for varying *Re* with constant w=1.5, r=0.75 and Pr=1. **(A)** Isotherm at *Re* = 500. **(B)** Isotherm at *Re* = 1000. **(C)** Isotherm at *Re* = 1500. **(D)** Isotherm at *Re* = 2000. **(E)** Isotherm at *Re* = 2500.

Fig 12 presents the isotherms for varying *Re* 500,1000, 1500, 2000 and 2500 while keeping *w* fixed at 1.5, *r* at 0.75 and *Pr* at 1.

At *Re* = 500, which is illustrated in Fig 12A, the heat transfer has subdued compared to higher *Re*. The right inlet, being moderately dominant, forms a vortex just after the smooth bend of the right wall of the outlet. This vortex slightly disturbs the thermal boundary layer, increasing heat transfer. Overall, at the outlet, the thermal boundary layer becomes thick, especially on the left side, which results in low heat transfer.

In Fig 12B, *Re* is taken 1000 and the isotherms show more noticeable distortion near the right wall of the outlet due to the intensified vortex. This causes the thermal boundary layer to thin at the right side of the outlet, increasing the heat transfer. On the left side, there is a slight increase in heat transfer. The thermal boundary layer is thick around the left wall of the outlet.

As *Re* increases to 1500, as shown in Fig 12C, as the vortex has increased in size and strength, it also leads to the secondary vortex. This results in more vigorous heat transfer enhancement across the outlet walls.

For higher *Re* of 2000 and 2500 Fig 12D and Fig 12E are illustrated. The fluid flow is unsteady, and the isotherms fluctuate with great complexity. This might be more controlled compared to Fig 11D and Fig 11E, where *r* was 0.25. The thermal boundary layer thickness has reduced. At all *Re*, the bottom wall shows the same behavior as *r* = 0.75. these vortices are relatively symmetric and weaker, resulting in modest thinning of the thermal boundary layer and a gradual enhancement of heat transfer

### Influence of *Re* on isotherms for *w* = 0.5

**Fig 13:**
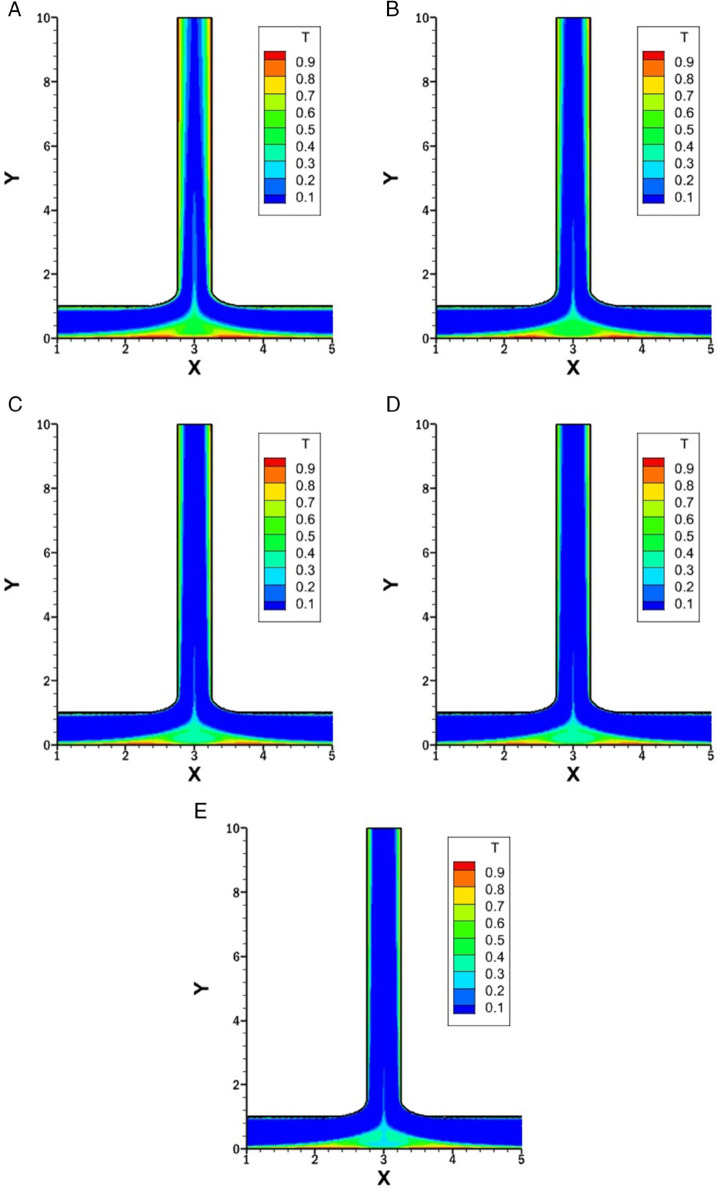
Isotherms for varying *Re* with constant w=0.5, r=1 and Pr=1. **(A)** Isotherm at *Re* = 500. **(B)** Isotherm at *Re* = 1000. **(B)** Isotherm at *Re* = 1500. **(D)** Isotherm at *Re* = 2000. **(E)** Isotherm at *Re* = 2500.

Fig 13, represents the isotherms for varying *Re* with constant *w* at 0.5, *Pr* at 1 and *r* at 1. These isotherms are symmetric as the flow rate ratio is symmetric leading to symmetric boundary condition. *w* = 0.5 shows that the outlet’s width is smaller than the inlets that causes the fluid to exit more rapidly, resembling a jet-like flow.

In Fig 13A at *Re* = 500, as there is no vortex along the outlet, the thermal boundary layer is thick which leads to reduced heat transfer. Along the bottom wall, as the flow from both inlets merge at the center, there is recirculation which makes the thermal boundary layer thin and enhance the heat transfer at this point.

As the *Re* increases to 1000 (Fig 13B) the only change observed is along the bottom wall, which is extremely small and due to which the thermal boundary layer along the outlet become more thick.

For *Re* = 1500 (Fig 13C), the overall pattern remains similar to *Re* = 1000 with only minor changes in the bottom wall recirculation region. The outlet boundary layer remains the same.

For *Re* = 2000 and *Re* = 2500 from Fig 13D and Fig 13E, the symmetry is preserved with no significant vortex development along the outlet. Heat transfer patterns are essentially unchanged from lower *Re* cases with weak enhancement only at the bottom wall recirculation point.

### Effect of *w* on isotherms for *r* = 0.5

**Fig 14:**
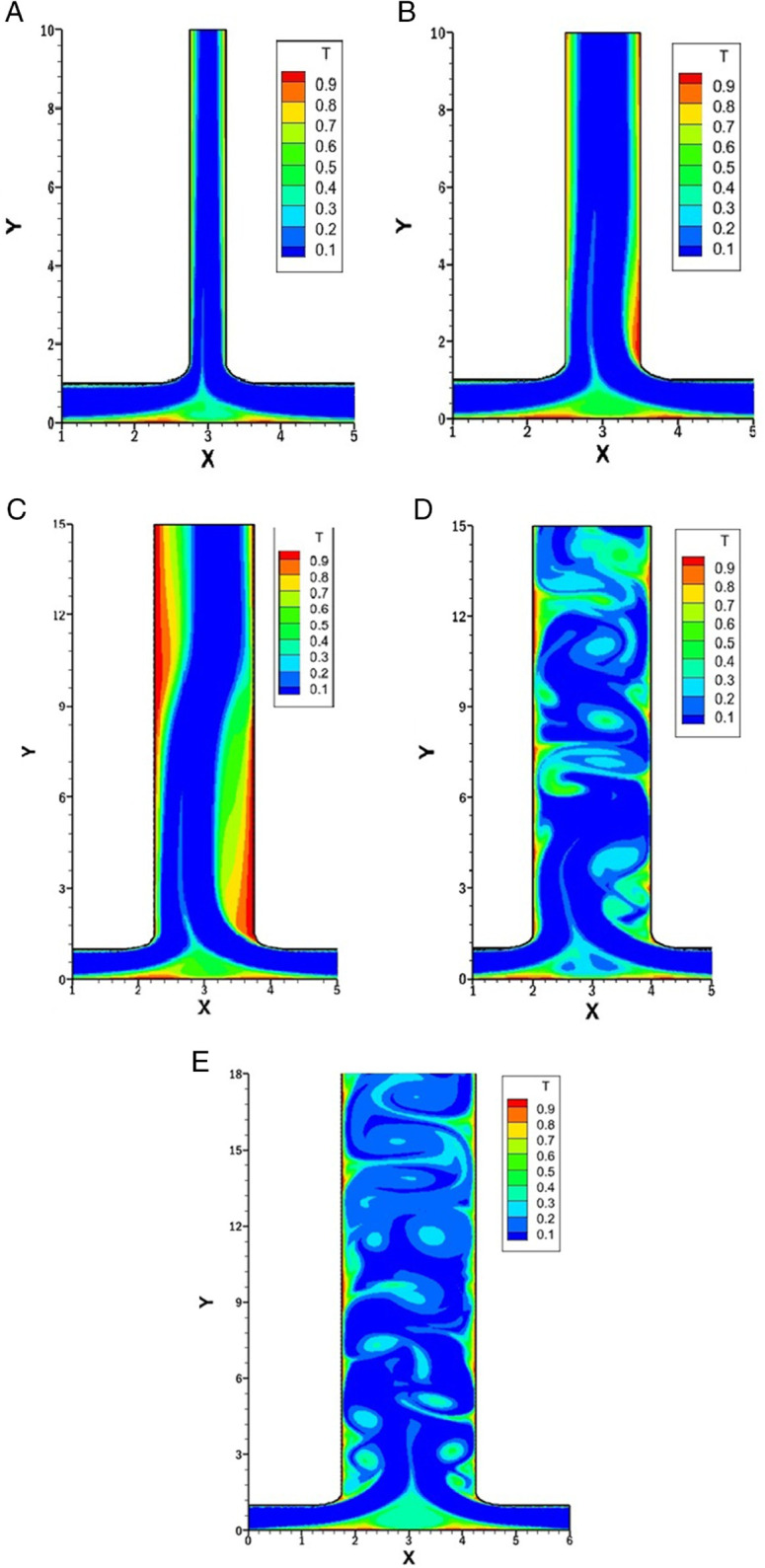
Isotherms for varying *w* with constant Re=1500, r=0.5 and Pr=1. **(A)** Isotherm at *w* = 0.5. **(B)** Isotherm at *w* = 1. **(C)** Isotherm at *w* = 1.5. **(D)** Isotherm at *w* = 2. **(E)** Isotherm at *w* = 2.5.

Fig 14 presents the isotherms for varying *w* at 0.5, 1, 1.5, 2 and 2.5 with fixed *Re* at 1500, *r* at 0.5 and *Pr* at 1.

In Fig 14A at *w* = 0.5, the outlet width is smaller than the inlets, forming a jet-like discharge. No vortices are present in the outlet region, and the thermal boundary layer remains thick, resulting in reduced heat transfer. The bottom wall exhibits thinning of the thermal boundary layer due to the merging of two inlets.

In Fig 14B, where *w* = 1, the outlet width matches the inlet length, providing a more balanced flow exit. A minimal vortex forms on the right side of the outlet, which locally disturbs the thermal boundary layer and slightly enhances heat transfer in that region. The left side of the outlet remains unaffected mainly, with a thicker thermal boundary layer.

In Fig 14C, where *w* = 1.5, a noticeable vortex is on the right wall of the outlet, which significantly disturbs the thermal boundary layer and increases the heat transfer. A secondary vortex is present on the left side of the outlet, which increases the heat transfer and thin the thermal boundary layer. The secondary vortex also helps in the mixing process.

At *w* = 2 and *w* = 2.5 in Fig 14D and Fig 14E, the outlets become extensively wider and the flow turns unsteady. This unsteady behavior causes the flow to fluctuate and mix more intensely throughout the outlet region. As a result, the thermal boundary layer becomes more disrupted on both sides, which improves heat transfer. The wider outlet promotes better fluid interaction, enhancing mixing and reducing the thermal boundary layer, which improves heat transfer.

### Effect of *r* on local Nusselt number for *w* = 1.5

**Fig 15:**
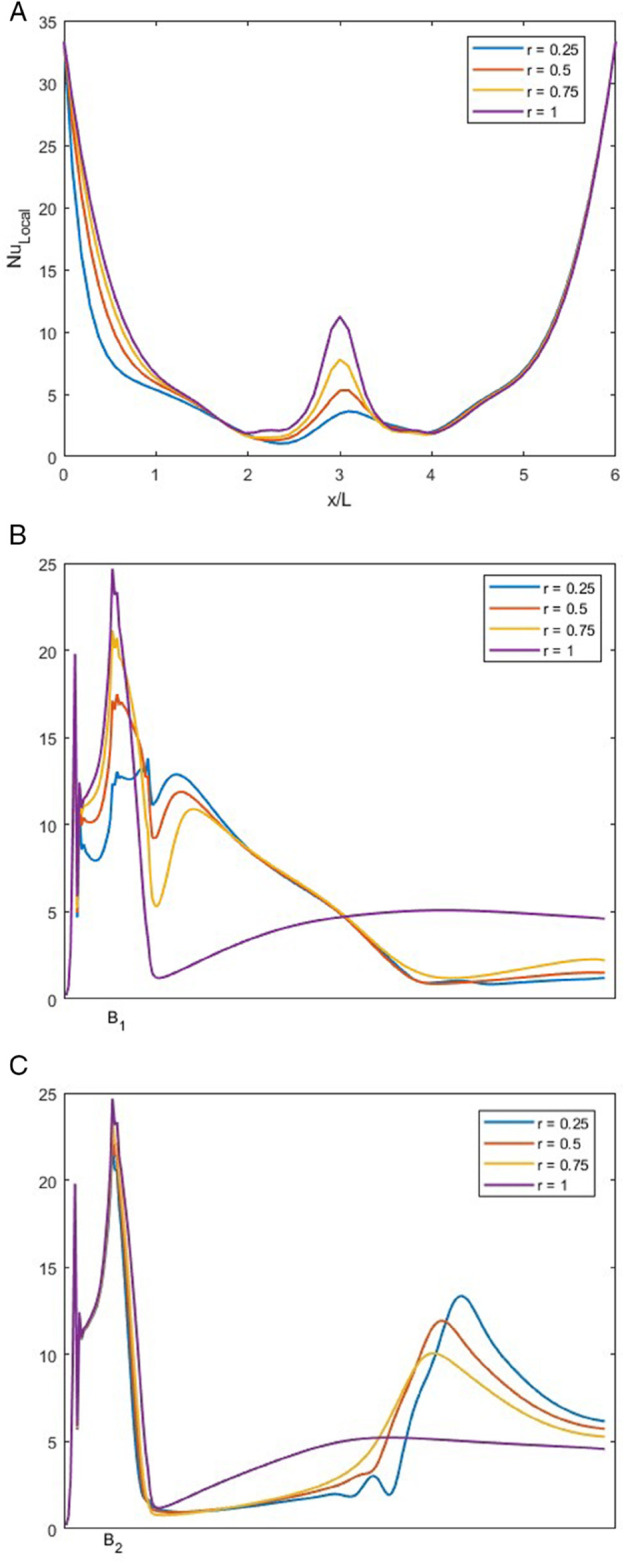
$Nu_{Local}$ for varying *r* with constant Re=1500, w=1.5 and Pr=1. (A) $Nu_{Local}$ along bottom wall of the junction. (B) $Nu_{Local}$ along the left wall of the junction. (C) $Nu_{Local}$ along the right wall of the junction.

Fig 15, $Nu_{Local}$ is plotted for varying *r* with constant *Re* at 1500, *w* at 1.5 and *Pr* at 1.

In Fig 15A, the $Nu_{Local}$ at the bottom wall is graphed. We can observe that the $Nu_{Local}$ along the bottom wall first decreases. This is due to the thick thermal boundary layer, as the flow reaches the center of the bottom wall, it collides with the flow of the opposite side and forms a vortex, due to this vortex, a sudden spike indicates enhanced heat transfer. This spike decays rapidly and the heat transfer decreases. $Nu_{Local}$ after the spike, a thick thermal boundary layer re-develops due to inflow from the right inlet. We can observe that as *r* increases, $Nu_{Local}$ also increases along the bottom wall.

In Fig 15B, $Nu_{Local}$ is plotted along the left outlet wall. As the fluid enters through the left inlet, a sharp spike in $Nu_{Local}$ is observed near the entrance. This is attributed to a small vortex forming at the start of the left outlet wall. For *r* = 0.25, 0.5, and 0.75, this vortex is relatively short in length, resulting in a less pronounced drop in $Nu_{Local}$ immediately afterward. However, for *r* = 1, the vortex is stronger and more symmetric, causing a significant initial spike followed by a notable decrease in $Nu_{Local}$ as the thermal boundary layer thickens. For the cases of *r* = 0.25, 0.5, and 0.75, a second spike is observed downstream, corresponding to the presence of a secondary vortex in the outlet channel, which enhances local heat transfer by disturbing the boundary layer.

In Fig 15C, $Nu_{Local}$ at right side is observed. A sharp increase in $Nu_{Local}$ is observed just after the bend of the smooth T-shaped junction, which is attributed to the presence of a strong vortex in that region. For all values of *r*, this initial rise in heat transfer is similar. However for *r* 0.25, 0.5 and 0.75 another increase in $Nu_{Local}$ occurs farther downstream, corresponding to the end of the vortex, where the thermal boundary layer is again disturbed.After this secondary enhancement, the flow stabilizes and $Nu_{Local}$ gradually settles as the boundary layer redevelops.

### Effect of *Re* on local Nusselt number at *r* = 0.25

**Fig 16:**
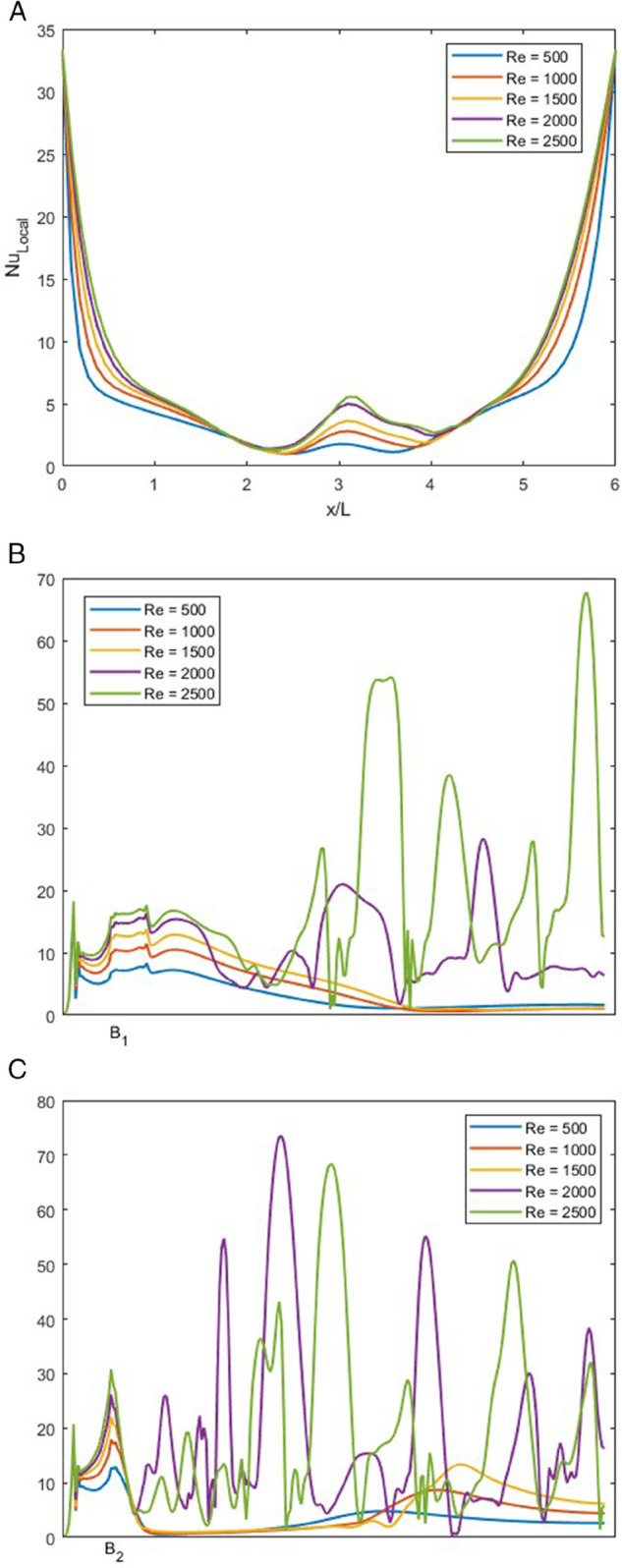
$Nu_{Local}$ for varying *Re* with constant r=0.25, w=1.5 and Pr=1. (A) $Nu_{Local}$ along bottom wall of the junction. (B) $Nu_{Local}$ along the left wall of the junction. (C) $Nu_{Local}$ along the right wall of the junction.

In Fig 16, $Nu_{Local}$ is illustrated for varying *Re* ranging from 500 to 2500 at fixed *r*, *Pr* and *w* of 0.25, 1 and 1.5, respectively.

In Fig 16A, $Nu_{Local}$ along the bottom wall is graphed. This trend is similar to Fig 15A, the difference is in the middle of the graph. As *r* is 0.25, the vortex present is pushed to the left, which makes the spike of $Nu_{Local}$ slightly off-center. We can observe this in each of the cases shown in Fig 16, whereas the Re increases, the intensity of $Nu_{Local}$ also increases.

In Fig 16B, we can observe that at the lower *Re* range from 500 to 1500, the $Nu_{Local}$ exhibits similar behavior but different intensities; the increase in $Nu_{Local}$ is where the vortices are present. For greater *Re*, as we have discussed in Fig 6D, the flow is unsteady. This unsteady behavior leads to chaotic behavior in the $Nu_{Local}$. We can observe that the $Nu_{Local}$ at the start of the vortices spikes and quickly decreases, but due to multiple vortices, the $Nu_{Local}$ can not settle.

In Fig 16C, we observe that as the fluid enters through the right inlet and when it reaches the smooth bend, there is a sudden increase in $Nu_{Local}$, which decreases almost immediately. For *Re* from 500 to 1500, we can see that it remains steady for some time, but after the vortex ends, it shows a slight peak and then settles down again. For higher *Re*, as the flow is unsteady, the behavior of $Nu_{Local}$ is also chaotic.

### Effect of *Re* on local Nusselt number at *r* = 0.75

**Fig 17:**
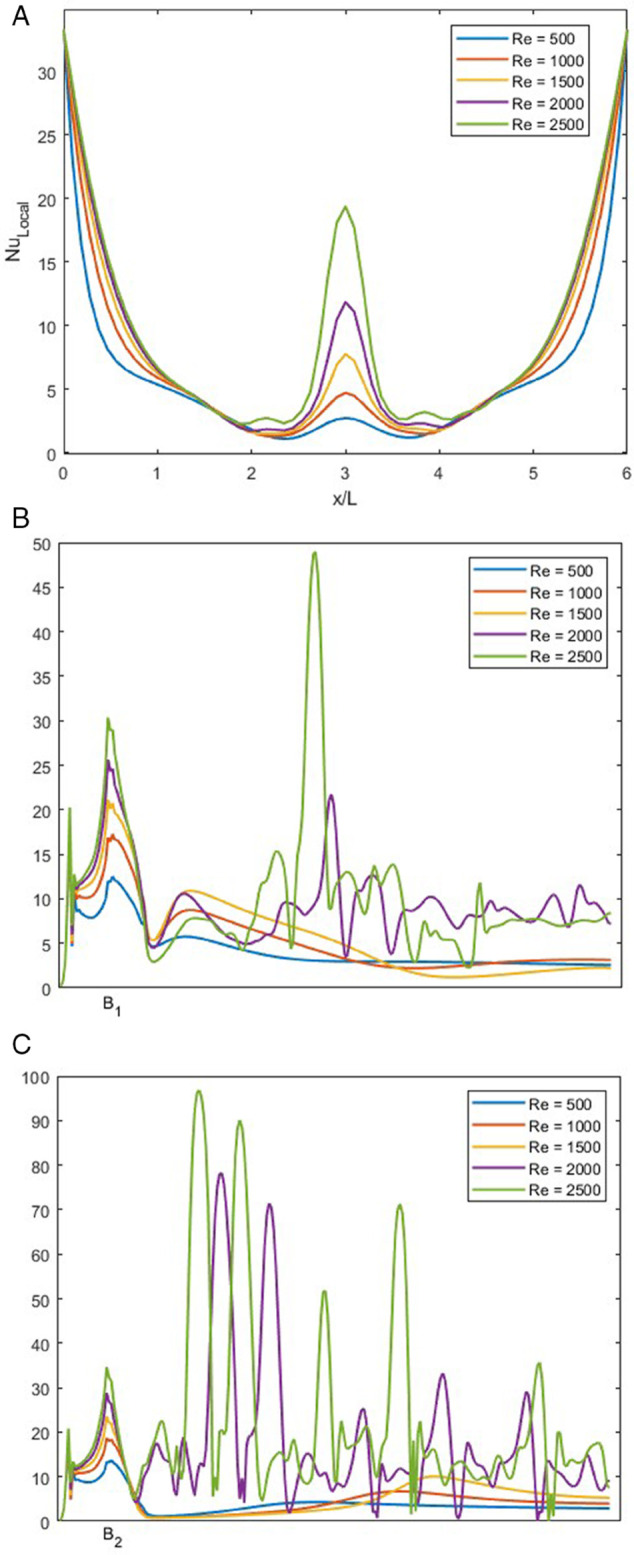
$Nu_{Local}$ for varying *Re* with constant r=0.75, w=1.5 and Pr=1. (A) $Nu_{Local}$ along bottom wall of the junction. (B) $Nu_{Local}$ along the left wall of the junction. (C) $Nu_{Local}$ along the right wall of the junction.

In Fig 17, $Nu_{Local}$ is illustrated for varying *Re* ranging from 500 to 2500 at fixed *r* = 0.75, *Pr* = 1 and *w* = 1.5.

In Fig 17A, we can observe the bottom wall, this figure has the same trend as in Fig 15A. As the fluid flow is more balanced than before, the spike is near the middle of the graph. Also, as the size of the vortex increases with the increase in the *Re*, the height of the spike increases.

In Fig 17B, we observe the left side of the T-shaped junction. As the fluid flows through the left inlet, the $Nu_{Local}$ observes a spike that quickly fades. At the end of the vortex, $Nu_{Local}$ again experiences a slight spike, but for *Re* values of 500 to 1500, it settles down along the left wall. For *Re* = 2000 and 2500, the $Nu_{Local}$ shows chaotic behavior, which is more intensified for *Re* = 2500.

In Fig 17C, we observe the $Nu_{Local}$ for the right side of the T-shaped junction. As the fluid reaches the smooth edge, it exhibits a spike, which corresponds to a local maximum for lower *Re* values. These $Nu_{Local}$ settles down along the left wall. For higher *Re*, we can again observe chaotic behavior, which is much more intense than that observed on the left wall.

### Effect of *Re* on local Nusselt number for *w* = 0.5

**Fig 18:**
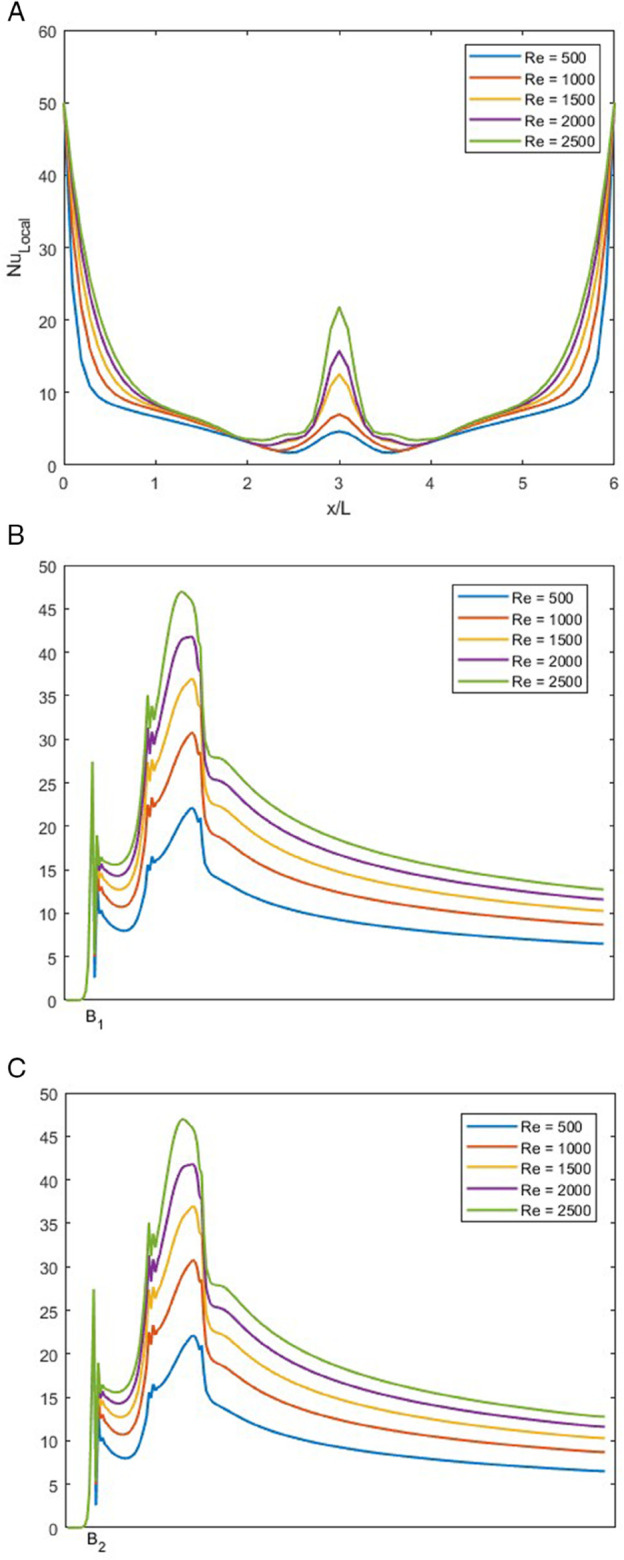
$Nu_{Local}$ for varying *Re* with constant r=1, w=0.5 and Pr=1. (A) $Nu_{Local}$ along bottom wall of the junction. (B) $Nu_{Local}$ along the left wall of the junction. (C) $Nu_{Local}$ along the right wall of the junction.

In Fig 18, $Nu_{Local}$ is illustrated for varying *Re* ranging from 500 to 2500 at fixed *r* = 1, *Pr* = 1 and *w* = 0.5.

In Fig 18A, the trend of $Nu_{Local}$ along the bottom wall of the T-shaped junction is illustrated. This trend is similar to the earlier bottom wall’s $Nu_{Local}$. The only difference is that the spike is directly in the center of the graph, as the flow rate ratio is symmetric. In Fig 18B and Fig 18C, we observe the symmetrical behavior. As both the trends are the same, we can analyze them more easily.

In Fig 18B, we observe that there is a spike in the behavior of $Nu_{Local}$ that is due to the change in the direction of the fluid flow. We can observe that after the spike, $Nu_{Local}$ begins to decrease in all values of *Re*. This is because $Nu_{Local}$ is lower when the thermal boundary layer is thicker.

In Fig 18C, the same trend as Fig 18B is observed. For all three graphs, the intensity of $Nu_{Local}$ increases with the increase in *Re*.

### Effect of *w* on local Nusselt number at *r* = 0.5

**Fig 19:**
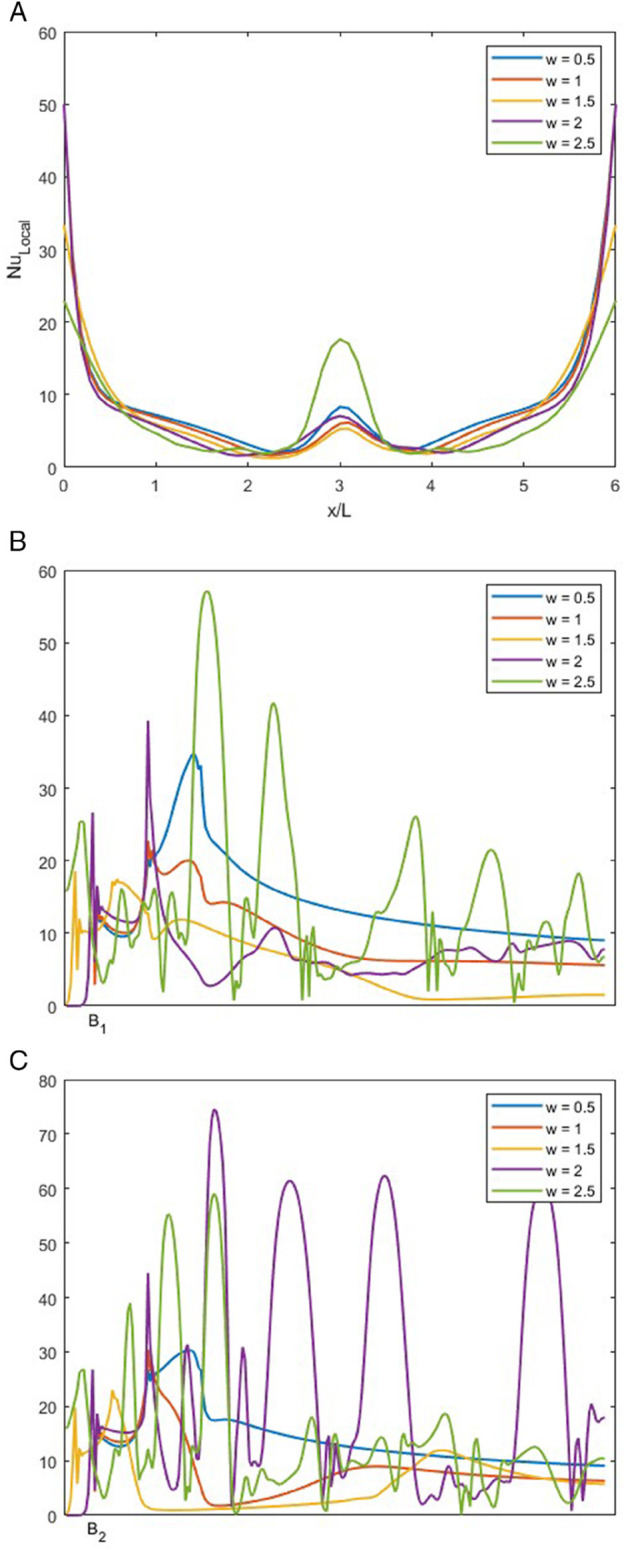
$Nu_{Local}$ for varying *w* with constant r=0.5, Re=1500 and Pr=1. (A) $Nu_{Local}$ along bottom wall. (B) $Nu_{Local}$ along the left wall. (C) $Nu_{Local}$ along the right wall.

In Fig 19, $Nu_{Local}$ is displayed for varying *w* at 0.5, 1, 1.5, 2 and 2 with fixed *r* = 0.5, *Re* = 1500 and *Pr* of 1.

In Fig 19A, we can observe the bottom wall’s $Nu_{Local}$ at different outlet regions. We observe that as the *w* increases, the $Nu_{Local}$ shows a variety of behaviors. Along the wall, $Nu_{Local}$ decreases, but at the point of recirculation, there is a spike in $Nu_{Local}$.

In Fig 19B, the behavior of $Nu_{Local}$ drastically from one *w* to another. At *w* = 0.5, the $Nu_{Local}$ exhibits a continuous spike that decreases and does not rise again, as there is no vortex in the outlet channel. For *w* = 1, the behavior is similar to *w* = 0.5 but less intense. For *w* = 1.5, we observe that the $Nu_{Local}$ spike is just in the beginning, but due to the presence of the vortex, it decreases immediately and keeps on decreasing. For *w* = 2, there are multiple smaller vortices along the left wall of the outlet, so there are sudden spikes in Fig 19B. For *w* = 2.5, compared to *w* = 2, the spikes are more intense, as the vortices are larger, indicating high heat mixing.

In Fig 19C, the trend of $Nu_{Local}$ varies for all values of *w*. For *w* = 0.5, 1, and 1.5, the $Nu_{Local}$ shows an increase at the bends of the smooth T-shape junction. For *w* = 2, as compared to the left wall, the vortices on the right wall are more intense, so the $Nu_{Local}$ here shows an intense behavior. It can be observed that after the initial vortices, the later vortices settle down. For *w* = 2.5, the behavior of $Nu_{Local}$ is purely chaotic, as along the right wall of the outlet, the vortices oscillate intensely.

The Table 9 presents Nusselt number values across the bottom, left, and right walls for varying *r*, *Re*, and *w*, with constant *Pr* = 1. When *r* increases (Fig 5), a gradual rise in $Nu_{avg}$ and $Nu_{min}$ is observed on all walls, while $Nu_{max}$ increases more noticeably, particularly on the outlet walls. As *Re* increases (Figs 6–8), the Nusselt numbers reflect stronger convection effects. Specifically, both $Nu_{avg}$ and $Nu_{max}$ show significant gains on the outlet walls, with higher enhancements observed at lower *r* (e.g., *r* = 0.25), where asymmetric flow induces strong thermal gradients. The bottom wall also shows a steady increase in $Nu_{avg}$, though $Nu_{max}$ remains fixed in some cases due to saturation or clipping. Lastly, when *w* varies (Fig 9), the Nusselt numbers reflect the influence of outlet width: narrower outlets (lower *w*) result in high local heat transfer near the center. In comparison, wider outlets allow more distributed thermal mixing, leading to fluctuations in $Nu_{max}$ and $Nu_{min}$ across all three walls.

**Table 9:** *Nu* along channel walls under varying parameters with Pr=1.

Index	Re	r	w	Bottom Wall			Left Wall			Right Wall		
				$Nu_{avg}$	$Nu_{max}$	$Nu_{min}$	$Nu_{avg}$	$Nu_{max}$	$Nu_{min}$	$Nu_{avg}$	$Nu_{max}$	$Nu_{min}$
1	1500	0.25	1.5	7.05	33.33	1.04	5.54	17.68	0.20	5.81	22.18	0.20
2	1500	0.50	1.5	7.64	33.33	1.30	5.79	18.53	0.20	5.91	22.98	0.20
3	1500	0.75	1.5	8.03	33.33	1.52	5.94	21.13	0.20	5.77	23.62	0.20
4	1500	1.00	1.5	8.49	33.33	1.87	5.56	24.68	0.20	5.59	24.67	0.20
5	500	0.25	1.5	5.48	33.33	1.00	3.29	16.75	0.20	3.54	17.96	0.20
6	1000	0.25	1.5	6.45	33.33	0.98	4.42	17.31	0.20	4.79	18.95	0.20
7	1500	0.25	1.5	7.05	33.33	1.04	5.54	17.68	0.20	5.81	22.18	0.20
8	2000	0.25	1.5	7.63	33.33	1.38	10.72	28.28	0.20	18.50	73.50	0.20
9	2500	0.25	1.5	7.89	33.33	1.31	19.08	67.70	0.20	18.34	68.35	0.20
10	500	0.75	1.5	6.16	33.33	1.12	4.27	17.68	0.20	4.03	17.97	0.20
11	1000	0.75	1.5	7.31	33.33	1.32	5.41	18.53	0.20	4.88	18.98	0.20
12	1500	0.75	1.5	8.03	33.33	1.52	5.94	21.13	0.20	5.77	23.62	0.20
13	2000	0.75	1.5	8.65	33.33	1.75	9.53	25.65	0.20	16.98	78.17	0.08
14	2500	0.75	1.5	9.59	33.33	2.30	10.43	48.97	0.20	22.41	96.87	0.05
15	500	1.00	0.5	7.37	50.00	1.70	9.68	25.62	0.00	9.68	25.62	0.00
16	1000	1.00	0.5	8.78	50.00	1.97	13.05	30.78	0.00	13.05	30.78	0.00
17	1500	1.00	0.5	10.08	50.00	2.73	15.53	36.97	0.00	15.53	36.97	0.00
18	2000	1.00	0.5	10.87	50.00	2.72	17.59	41.83	0.00	17.59	41.83	0.00
19	2500	1.00	0.5	11.87	50.00	3.40	19.40	46.93	0.00	19.40	46.93	0.00
20	1500	0.50	0.5	9.13	50.00	2.08	13.68	34.69	0.00	13.62	30.26	0.00
21	1500	0.50	1.0	8.49	50.00	1.66	9.12	26.21	0.00	8.20	30.41	0.00
22	1500	0.50	1.5	7.64	33.33	1.30	5.79	18.53	0.20	5.91	22.98	0.20
23	1500	0.50	2.0	7.83	50.00	1.61	7.55	39.26	0.00	23.66	74.56	0.00
24	1500	0.50	2.5	7.13	22.92	1.88	14.91	57.16	0.41	13.77	59.04	0.13

## 4 Conclusion

Our study represents a detailed numerical investigation of fluid flow and heat transfer in a two-dimensional T-shaped junction with smooth bends. The simulations were conducted utilizing the finite difference method, IMEX method and employing compact upwind schemes for improved accuracy. The simulations were conducted in MATLAB software. The analysis considers a wide range of Reynolds numbers (*Re* = 500 to 2500), volumetric flow rate ratios (r=0.25,0.5,0.75,1), Prandtl numbers (*Pr* = 1), and outlet cross-sectional width ratios (w=0.5,1,1.5,2,2.5). The fluid flow behavior is studied through streamlines and heat transfer performance is evaluated using isotherm distributions, along with detailed Nusselt number data for the bottom, left, and right walls. The findings offer valuable insight into thermal performance in T-junction geometries. The key conclusions are:

- When the value of *r* increases, the flow moves from unbalanced to balanced flow. The size and intensity of the vortices decreases Also the secondary vortex weakens. For *r* = 1 symmetric behavior of streamlines is observed.
- When the value of *r* increases, the thermal boundary layer become thin along the outlet which results in less heat transfer.
- For increasing *r*, a steady increase in both $Nu_{Local}$ and $Nu_{Avg}$ is observed along the walls indicating better heat transfer performance with balanced flow.
- For both *r* = 0.25 and *r* = 0.75 with fixed *w* = 1.5, increasing the value of *Re* from 500 to 1500 increases the size of the vortices and also creates the secondary vortices. For higher Re≥2000, flow becomes unsteady with chaotic oscillations.
- For increasing *Re* from 500 to 1500 at *r* = 0.25 and 0.75, the heat transfer along the outlet increases making the thermal boundary layer thin. For Re≥2000, due to multiple interacting vortices intensified thermal mixing is observed, making this analysis more complex.
- $Nu_{\text{Local}}$ plots confirm the emergence of localized spikes in heat transfer where vortices interact with the walls.
- At small *w* = 0.5, the outlet behaves like a jet, preventing vortex formation along the outlet walls = which leads to thick thermal boundary layer with weak heat transfer.
- As the *w* increases, vortices begin to form along the outlet walls which enhances the heat transfer, $Nu_{Local}$ and $Nu_{Avg}$.
- As w≥2, the flow become unsteady resulting in chaotic behavior and fluctuation which increases the heat transfer and disturbs the thermal boundary.
- The presence of strong primary and secondary vortices leads to sharp spikes in $Nu_{\text{Local}}$, highlighting zones of intense heat exchange.
- Unsteady flows exhibit fluctuating $Nu_{\text{Local}}$ profiles, which although harder to predict, provide superior thermal performance.
